# Linking Labile Heme with Thrombosis

**DOI:** 10.3390/jcm10030427

**Published:** 2021-01-22

**Authors:** Marie-Thérèse Hopp, Diana Imhof

**Affiliations:** Pharmaceutical Biochemistry and Bioanalytics, University of Bonn, An der Immenburg 4, 53121 Bonn, Germany; mhopp@uni-bonn.de

**Keywords:** blood coagulation, coagulation factors, heme binding, hemolysis, hemolytic diseases, hemorrhage, labile heme, platelets, thrombosis

## Abstract

Thrombosis is one of the leading causes of death worldwide. As such, it also occurs as one of the major complications in hemolytic diseases, like hemolytic uremic syndrome, hemorrhage and sickle cell disease. Under these conditions, red blood cell lysis finally leads to the release of large amounts of labile heme into the vascular compartment. This, in turn, can trigger oxidative stress and proinflammatory reactions. Moreover, the heme-induced activation of the blood coagulation system was suggested as a mechanism for the initiation of thrombotic events under hemolytic conditions. Studies of heme infusion and subsequent thrombotic reactions support this assumption. Furthermore, several direct effects of heme on different cellular and protein components of the blood coagulation system were reported. However, these effects are controversially discussed or not yet fully understood. This review summarizes the existing reports on heme and its interference in coagulation processes, emphasizing the relevance of considering heme in the context of the treatment of thrombosis in patients with hemolytic disorders.

## 1. Introduction

Worldwide, one in four people die from cardiovascular diseases related to thrombosis [1]. In the case of thrombosis, an imbalance of blood coagulation occurs, leading to the formation of a blood clot, and consequently to partial or complete vascular occlusion [2]. Whether arterial or venous, a blockage in the vasculature causes a limitation of the blood flow, which usually relies on a complex homeostatic interplay of plasma proteins (i.e., coagulation and inflammatory factors), blood cells (in particular platelets and red blood cells (RBCs)) and the endothelium [2,3]. While arterial thrombosis, such as in atherosclerosis, commonly follows the rupture of an atheroma under conditions of high shear stress, venous thrombosis, such as deep vein thrombosis (DVT), occurs mostly at sites of intact endothelium under low shear stress conditions [4,5,6]. Apart from inherited coagulation disorders, major risk factors for thrombotic abnormalities are for example age, life-style factors, such as smoking and obesity, as well as trauma and surgeries [7,8,9,10]. As such, thrombotic events contribute to a variety of clinical sequelae encompassing ischemic heart disease and stroke [2,6,11]. Moreover, several hemolytic conditions, such as sickle cell disease (SCD) [12,13,14,15,16,17,18,19,20,21,22,23,24], hemolytic uremic syndrome [25,26], hemoglobinuria [27,28], hemolytic transfusion reactions [29,30,31,32,33,34,35,36], hemorrhage [33,37,38,39,40,41,42], and cardiac surgery [43,44], were reported to manifest thrombotic complications as thrombosis, hypercoagulability, and vasculopathy [45,46,47,48,49,50,51,52,53,54,55,56]. In adult SCD patients, venous thromboembolism (VTE) is even one of the leading causes of death [57,58]. The thrombophilic status of patients is likely driven by multiple factors. Therefore, the following changes in hemostatic biomarkers are striking: Elevated levels and/or activity of clotting proteins (e.g., tissue factor (TF) [59,60], factor VIII (FVIII) [45,61,62], fibrinogen [45]), reduced levels of contact proteins (e.g., factor XII (FXII) [63], prekallikrein [63,64], high molecular weight kininogen [63,65]), increased levels of fibrinolytic markers (e.g., D-dimers [28,66,67], fibrinopeptide A [68,69], plasmin-antiplasmin complexes [16,67], fibrinogen-fibrin degradation products (FDP) [70,71]), increased expression of adhesion proteins (e.g., intercellular adhesion molecule 1 (ICAM-1) [72,73], vascular cell adhesion molecule 1 (VCAM-1) [72,74], von Willebrand factor (VWF) [75,76], P-selectin [28,74,77], thrombomodulin [78,79]), decreased levels and/or activity of anticoagulant proteins (e.g., protein C [68,80,81,82,83], protein S [68,80,81,82], antithrombin [84]), and an increase of platelet activation [85,86]. In addition, exposure of negatively charged phosphatidylserine on cell surfaces is increased providing a binding site for accumulated enzyme complexes, which further enhances procoagulant activity through support of thrombin generation and platelet activation [19,87,88]. However, the concrete underlying mechanism is still not entirely known [48,50].

These hemolytic disorders are associated with an excessive release of labile heme, which itself is capable of exerting a great variety of functions [89,90,91,92]. As a permanently bound prosthetic group, heme equips numerous hemoproteins with their different properties [92,93]. The vital role of heme in the cardiovascular system can be demonstrated using the examples of oxygen transport by hemoglobin, antioxidant activity of peroxidases and catalases or electron transfer by cytochromes [92,93,94]. Moreover, heme is well-known as a signaling molecule that acts through transient binding to various proteins mediating a wide range of biochemical processes, such as transcription, inflammation, or signal transduction [90,91,92].

In the human body, 80% of heme is produced and found in RBCs, of which there are up to 25 trillion occurring in the circulation [90,95]. Therefore, the concentration of extracellular heme is relatively low under physiological conditions [92,96]. In hemolytic situations, extracellular labile heme levels can reach dangerously high concentrations [97,98]. While extravascular hemolysis results in the phagocytosis of RBCs by macrophages of the reticuloendothelial system (RES) especially in the liver and the spleen, intravascular hemolysis leads to the lysis of RBCs and release of the RBC content into the vascular compartment [99,100,101]. The damage of each RBC can result in the release of about 2.5 × 10^8^ hemoglobin molecules from a single RBC into the bloodstream [102]. After dissociation into αβ dimers, free hemoglobin is scavenged by the acute phase serum protein haptoglobin, thereby preventing from hemoglobin-mediated oxidative damage, renal infiltration, and iron loading [96,103,104,105,106]. Subsequently, the complex is recognized by the macrophage-specific surface protein cluster of differentiation (CD) 163, which arranges the uptake into macrophages of the RES and, thus, the clearance of both, hemoglobin and haptoglobin, from circulation by lysosomal breakdown to heme, peptides and amino acids [107,108,109,110]. The resulting heme is sequestered by the heme oxygenase system yielding biliverdin, iron ions, and carbon monoxide [107,111,112]. Heme oxygenase 1 (HO-1) expression can be induced by heme itself, which consequently prevents from harmful effects like oxidative stress, inflammation or ischemia-reperfusion injury [113,114]. However, in case of more extensive hemolysis, the hemoglobin-binding capacity of haptoglobin becomes overwhelmed, followed by the accumulation of hemoglobin in plasma [107,114]. Hemoglobin is then oxidized to methemoglobin [115,116], which results in the release of large amounts of labile heme [117,118,119]. Consequently, heme is scavenged by different plasma proteins, in particular hemopexin, α1-microglobulin, albumin, and lipoproteins, and eventually detoxified through the formation of non-toxic heme-protein complexes [96,99,113]. First, liberated heme is mainly bound to albumin as the most abundant protein in plasma (~300 µM) possessing a low affinity (K_D_ ~40 µM) and a high affinity binding site (K_D_ ~20 nM) for heme [120,121]. Subsequently, it is transferred to hemopexin, which is known as the heme-binding protein with the highest heme-binding affinity (K_D_ ~5.3 fM) [122,123]. The plasma level of hemopexin (~20 µM) is considerably lower than of that of albumin, explaining the initial association of heme with albumin [110]. Hemopexin then transports heme to the parenchymal liver cells, where the internalization of the heme-hemopexin complex is directed by the low-density lipoprotein receptor-related protein 1 (LRP1)/CD91, which results in the cellular uptake and degradation of heme [99,124]. Nevertheless, in severe hemolytic states, such as occurring in sickle cell disease, ischemia-reperfusion injury or blood transfusion [125,126,127], these heme detoxification systems become overwhelmed. In this case, excess of heme can lead to toxic effects, evoking oxidative stress, inflammation and hemolysis [94,128,129]. Due to the redox-active nature of the iron ion in labile heme, its toxicity is mainly based on the heme-induced generation of reactive oxygen species (ROS), leading to damage of lipid membranes, nucleic acids, and proteins by Fenton reaction and subsequently to cellular injury and cell death [113,130,131,132]. In addition, heme-mediated ROS production and membrane damage of RBCs can induce hemolysis, which, in turn, leads to a further increase of extracellular heme levels [113,131]. Heme has also been recognized as a damage/danger-associated molecular pattern (DAMP) triggering a large number of proinflammatory pathways (e.g., nuclear factor kappa B (NF-κB), activator protein 1 (AP-1), and specificity protein 1 (SP-1) signaling) [129,133].

Therefore, it is not surprising that patients with any diseases accompanied by hemolysis suffer from heme toxicity related symptoms, in particular acute proinflammatory responses [98,134,135,136]. A role of heme in the induction and mediation of the thrombotic effects accompanying hemolysis is discussed as well [97,98,137]. Here, we review the role of heme and its interactions with components of the blood coagulation system and discuss the implications of heme in initiating and processing thrombosis of patients with hemolytic diseases.

## 2. Thrombotic Complications upon Heme Injection

First trials to study the effect of hematin (ferri(Fe^3+^)heme hydroxide) injection can be traced back to the year 1911 [138], shortly before the structure elucidation of hemin (ferriheme chloride) was completed by W. Küster (1912) [139]. Actually, the study aimed at deciphering the origin of hematosiderin (an iron-storing complex) and bilirubin (“hematoidin”) from hematin as a potential source and suggested, for the first time, its release from degraded hemoglobin from ruptured RBCs [138]. Experiments with intravenous and intraperitoneal injection of an alkaline hematin solution (30–54 mg/kg) in guinea pigs and rabbits, respectively, had to be abandoned due to the extreme toxicity of the solution. An autopsy after subcutaneous and intraperitoneal injection of hematin and hemin, both suspended in salt solution at a concentration of 30 mg/kg produced hemorrhage and ecchymosis, an extravasating bleeding usually associated with small vessel lesions in subcutaneous tissues [138]. Moreover, a pronounced tendency towards hemorrhage was observed in the affected and surrounding tissue [138]. In 1913, this was confirmed in cats and dogs after injection of hematin (in the range of 3.5–9 mg per kg body weight) into the viscera and the peritoneal cavity [140], as well as in rabbits, where it occurred as hemorrhagic kidney injury and/or extensive hemorrhage in the peritoneal cavity after injection of large hematin doses (10–25 mg per kg) [141,142]. Furthermore, W. H. Brown described the formation of hyaline thrombi or emboli in the smaller, primarily glomerular vessels and subsequent occurrence of vaso-occlusion and vascular injuries. In some cases, even infarcts were observed [141]. This study thus provided a first indication of heme-induced thrombotic complications (Supplementary Table S1). About 30 years later, Anderson and colleagues injected hematin (i.a., 200 mg intraperitoneally and 20 mg subcutaneously) in dogs and confirmed these observations [143]. They noted, in particular upon intravenous injection, conspicuous changes of the vasculature, comprising congestion, hemorrhage, and thrombosis, especially in small vessels. In addition, the authors stated that the observed effects were highly similar to those usually occurring under hemolytic conditions [143]. In contrast, Corcoran and Page described in 1945, among other symptoms, an anticoagulant effect after hematin injection into dogs, resulting in an inhibition of the coagulation process [144]. In 1966, Gessler and coworkers were faced with massive hemorrhagic bleeding in rats after injection of hematin (100–180 mg per kg body weight). The rats died a few seconds after injection, while bleeding from every possible body orifice [145].

As the aforementioned studies were mostly performed in view of the characterization of the malarial pigment (today known as the crystallized form of heme “hemozoin”), in 1971 researchers started to consider the therapeutic use of heme in the context of porphyria treatment [146]. Therefore, more experiments for the clarification of heme toxicity were undertaken (Supplementary Table S1) [147]. Hemin dissolved in 1% Na_2_CO_3_ was intravenously administered in rats (30–60 mg per kg body weight). Thereby, most of the rats (88.9%) that received the lowest dose (30 mg hematin per kg body weight) stayed alive. After injection of 40 mg hematin per kg body weight, for 30% of the rats in the experimental group internal bleeding was discovered postmortem [147]. With the highest dose (60 mg per 1 kg body weight) applied, 100% of the rats died. As previously observed, bleeding into small intestine and petechiae of liver, lungs and adrenals were found. In addition, hemorrhage and subcutaneous hematomas were detected. The LD_50_ was determined to be 43.2 mg hematin per kg body weight [147]. At that time, porphyria patients (acute intermittent porphyria (AIP), porphyria variegate or hereditary coproporphyria) were already successfully treated with hematin [148,149]. The administered dose of 4 mg/kg was effective and reported without any negative side effects, suggesting the relevance of coagulopathies as a consequence of heme injection only upon administration of excessive amounts [147,148]. Therefore, hematin was introduced as a drug (Panhematin^®^) in the USA in 1983 [150].

In contrast, in 1975 Dhar et al. observed thrombophlebitis after hematin infusion (prepared in alkaline solution, 1.2–6 mg/kg) in patients with hepatic porphyria [151]. Few years later, Lamon et al. reported chemical phlebitis at the site of hematin infusion in some cases, although less hematin was administered (2 mg/kg; reconstituted in saline solution) [152]. Here, the infusion was organized in a consecutive, daily manner, while applying hematin (2 mg/mL in saline solution) over a time period of 15 minutes (min) as the maximum infusion time [152]. In 1981, Morris and coworkers confirmed these observations in a patient with AIP. Here, 196 mg hematin was intravenously injected every 12 hours (h). Afterwards, pronounced coagulation was determined, without an explanation of the underlying mechanism [153]. In the following years, more and more studies supported the procoagulant effect of hematin as evidenced by thrombophlebitis. In the study of McColl et al., hematin was prepared in a 1% Na_2_CO_3_ solution and stored at 4 °C up to 10 days before use [154]. A dose of 4 mg/kg was intravenously infused in patients with acute porphyria attacks either every 12 h or as a daily dose. As a consequence of the injection in a small peripheral vein, phlebitis arose in up to 30% of the patients, in two cases even severe phlebitis [154]. Sometimes comparable hematin doses (3–4 mg/kg) resulted in the formation of bile thrombi [155]. These thrombophilic reactions were reported not only in patients with porphyria, but also in healthy volunteers (in 45% of the cases) upon heme administration (4 mg/kg body weight) [156]. Thrombophlebitis was limited to the vein in which hematin was infused. In patients without thrombophlebitic reaction, fibrotic events as well as vaso-occlusion were observed [156]. In contrast, hemostatic parameters suggested an anticoagulant role of hematin (Section 3) [156].

Since these observations were not uniform, varying from mild to severe effects, as well as from bleeding to thrombotic events, Goetsch and Bissell suggested the instability of hematin as the major cause for these differences [157]. In 1988, Simionatto et al. correlated hemostatic parameters with the actual hematin concentration in the plasma of nine test persons. They found a 30% loss of hematin in plasma, which might be an evidence for the conversion into related degradation products [156]. In addition, it might be also an indication for the instability of the hematin solutions, conceivably responsible for the thrombotic effects [156]. Due to the instability of hematin there were efforts deployed to improve its effectiveness and reduce adverse effects. In 1987, Tenhunen and coworkers presented for the first time a stable, administrable heme compound, so-called heme arginate (later approved as drug “Normosang^®^”) [158]. The compound was prepared as hemin arginate (corresponding to 25 mg/mL hemin) in an aqueous solution with 40% 1,2-propanediol and 10% ethanol added. Even after repetitive infusion of heme arginate (5 mg/kg) in rabbits there were no side effects observed, in particular no thrombophlebitis or other signs of prothrombotic states. In comparison, the administration of hematin (in form of the drug “Panhematin^®^”) resulted again in thrombophlebitic events [158]. In mice, the LD_50_ for heme arginate was determined to be 56.3 mg/kg (intravenous administration), 112.5 mg (intraperitoneal administration) and >5 g (oral administration) [158]. Therefore, the LD_50_ of heme arginate is fundamentally higher than for hematin [147,158]. Clinical trials with heme arginate (Normosang^®^) were already started at that time, forming the basis for the introduction as a drug in the European pharmaceutical market [158]. The higher safety with heme arginate treatment might be due to the fact that it is not as potent as heme in catalyzing free radical reactions and thus sensitizes endothelial cells to oxidant injury to a lesser extent [159,160]. Nevertheless, thrombophlebitic or bleeding effects occasionally appeared in humans as a complication as well (Supplementary Table S1) [161,162,163,164,165]. In another approach, heme complexed with albumin was administered [166]. The compound, consisting of 0.5 mM heme and 0.5 mM albumin, was well-tolerated in all test persons and showed no evidence of instability. Thereby, neither thrombosis nor bleeding was observed, probably because of complexation of heme with albumin [166,167].

In the USA, Panhematin^®^ was and still is the only approved heme-based drug and, thus, the compound of choice. Therefore, physicians are still faced with the abovementioned disturbances of the hemostatic system in the context of heme therapy in porphyrias. In 2000, Gajra and coworkers reported another case of an AIP patient that developed clinically obvious coagulopathy after hematin (Panhematin^®^) treatment as a single dose of 4 mg/kg [168]. The patient recovered 72 h after treatment. More recently, in the broad study of Anderson and Collins reported in 2006 it was found that 3.1% of the treated patients showed thrombotic complications as side effects after hematin administration [150].

**Figure 1:**
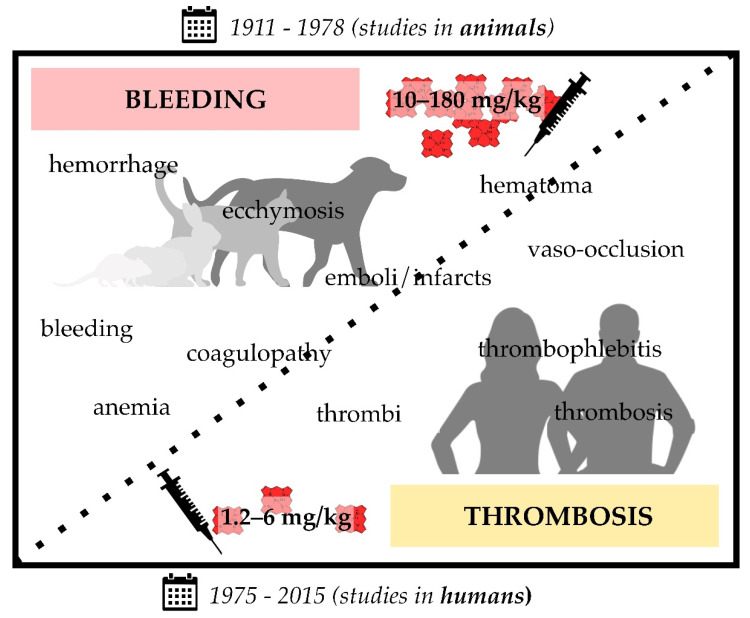
Infusion of heme in different formulations (i.e., hematin, hemin, heme arginate (Normosang^®^), Panhematin^®^) leads to coagulation disorders in animals (1911–1978; rats, guinea pigs, rabbits, cats, and dogs) and humans (1975–2015). Heme infusions of rather high concentrations (10–180 mg/kg) were shown to cause bleeding symptoms in animals, such as hemorrhage and coagulopathy, whereas administration of comparatively low concentrations (1.2–6 mg/kg) resulted in prothrombotic symptoms, such as vaso-occlusion and thrombophlebitis. The transition between bleeding and thrombotic events seems to be smooth, since there are also studies that report both effects in the same study (10–25 mg/kg hematin) [141,143]. Moreover, there are single exceptions (three studies) that also report bleeding upon administration of lower hematin (3.5–9 mg/kg) [140,162] or heme arginate (2–3 mg/kg) [168] concentrations.

Taken together, there were bleedings and hemorrhages observed, partially postmortem, when animals were treated with immense doses (14–180 mg/kg) of hematin or hemin (Figure 1, Supplementary Table S1). In addition, upon administration of hematin in the range of 10–25 mg/kg, both, hemorrhage and thrombotic complications, occurred. In humans, either healthy or porphyria patients, considerably lower concentrations of hematin or heme arginate were injected, resulting in thrombotic complications. The only exceptions are a report on the infusion of 2–3 mg/kg heme arginate, which led to occasional bleeding in patients with myelodysplastic syndrome [162] as well as the description of coagulopathies and hematomas in an AIP patient who was treated with Panhematin^®^ (Supplementary Table S1) [168].

Based on these observations, authors recommended complete monitoring of coagulation parameters, such as clotting times and platelet count, during hematin therapy in porphyria patients [153,168,169].

## 3. Heme-Mediated Interference in Coagulation Point-of-Care Testing

Since screening of coagulation factor levels is usually time consuming and appropriate tests are of limited availability, various routine coagulation tests are employed conventionally in order to characterize the hemostatic state of patients. Today, the determination of the activated partial thromboplastin time (aPTT) and the prothrombin time (PT) are common procedures. The aPTT is usually used to control the proper course of the intrinsic and common pathway of coagulation. Citrated plasma is mixed with phospholipids and a contact activator, such as Kaolin [170]. Consequently, factor XI is activated, but only when calcium ions are added the coagulation can proceed. The time to complete clotting is then recorded as aPTT. Clotting factor deficiency (i.e., factor (F)I, FII, FV, FVIII, FIX, FXI, FXII, high-molecular-weight kallikrein and kallikrein) or an impaired activity of the same results in an aPTT prolongation [170]. In contrast to the aPTT, PT allows for the evaluation of the extrinsic and common coagulation pathway. Thus, first TF is added to activate the extrinsic pathway, and then calcium ions. From prolonged PT, impaired levels or activity of the following clotting factors can be derived: FI, FII, FV, FVII, and FX [171]. To enable a comparison between the results from different laboratories, sometimes a standardized prothrombin time ratio, the international normalized ratio (INR), is determined [172]. Among aPTT and PT screening assays, other clotting and bleeding times that were analyzed in the past and/or still today are the following: Thrombin time (TT; fibrinogen-dependent), fibrinogen time (FT; fibrinogen-dependent), reptilase time (RT; fibrinogen-dependent), bleeding time (platelet-dependent), and the ethanol gelation test (fibrinogen-dependent). These were also used to characterize the effect of heme in its different formulations on the coagulation process in vivo and in vitro (Supplementary Table S2).

Already in 1913, a prolongation of the bleeding time (Duke method) was observed as a consequence of hematin injection (25 mg/kg) in rabbits [142]. The animals continued bleeding for several hours (>2 h) after a small cut into the ear. In consent with these observations, Brown et al. showed that hematin (25 mg/kg) was capable of prolonging the coagulation time in rabbits from an average value of 8–11 s to 17 s [142]. In the same setting, prolongation of the bleeding time was more pronounced. Indeed, Barnard (1947) observed a similar, concentration-dependent effect on the TT by adding 20–60 mg.% lithium ferriheme to plasma samples, even up to a complete loss of clotting ability (Supplementary Table S2) [173]. The author suggested that this might be due to heme interaction with for example thiol groups that might play a role in blood coagulation. In contrast, lithium ferriheme did not induce any significant changes of TT [173].

About 35 years later, Morris and colleagues treated a female AIP patient with hematin (196 mg, every 12 h) and observed markedly prolonged PT (from 13.2 s to 20.2 s), as determined 11 h after the first hematin administration [153]. The same was realized for the aPTT (from 25–41 s to more than 60 s), but no sign of thrombophlebitis occurred [153]. Upon hematin infusion (4 mg/kg, every 12 h) in an AIP patient, Glueck et al. confirmed the previous observations with both, an increase of the PT (from 11.7 s to 18.3 s) and a marked prolongation of the aPTT (from 37.5 s to more than 150 s) [169]. In contrast, the TT stayed unaltered. Based on these observations, the group continued with in vivo studies, focusing on the effect of hematin on the PT, aPTT, TT, and RT. For this purpose, 4 mg/kg hematin was administered by intravenous infusion over a period of 15 min, blood samples were taken over a time range of 0–48 h after infusion and subsequently processed for hemostastic characterization by clotting times. Already 10 min after infusion all parameters were extended in the collected samples: The aPTT increased from ~30 s to ~100 s, the PT from ~4 s to ~10 s, the TT from ~10 s to ~25 s, and the RT from 20 s to 30 s [169]. In a second approach, the same patient was pretreated either with 650 mg acetylsalicylic acid or with 5000 U heparin 2 h and 10 min, respectively, before hematin infusion (4 mg/kg). Interestingly, although acetylsalicylic acid and heparin are already potent anticoagulant compounds, the addition of hematin still increased the effect on the clotting times in comparison to the acetylsalicylic acid and heparin baseline [169]. However, in all approaches the coagulation parameters returned to normal levels (latest 48 h after hematin injection), with hematin levels also normalized [169]. In vitro, hematin (0.01 mg/mL and 0.1 mg/mL, respectively) prolonged the TT and RT of normal plasma as well. In the same year, Green et al. confirmed that hematin (0.01 mg/mL) is capable of prolonging the TT (from ~13 s to ~46 s) [174]. Upon administration of hematin in complex with albumin a fourfold concentration of hematin was necessary to induce a prolongation of the TT [174]. In contrast, Morris et al. did not detect bleeding in an AIP patient after hematin treatment (196 mg, every 12 h) [153].

Shortly thereafter, it became doubtful whether or not the observed effects were induced by hematin itself [156,157,175]. Goetsch and Bissell (1986) compared the PT of plasma in the presence of fresh and aged hematin (40 mg/L) in vitro [157]. While freshly prepared hematin (in 0.1 M Na_2_CO_3_, pH 8.0) did not affect the PT, stored hematin slightly prolonged this clotting time in vitro. This effect was more pronounced when hematin was stored longer and at room temperature (e.g., after 50 h of storage: ~16.3 s) instead of storage at 4 °C (e.g., after 50 h storage: ~15.8 s). Based on these results the authors recommended the use of freshly prepared hematin solution for infusion in the treatment of AIP, in order to prevent coagulopathies as a side effect of hematin administration [157]. In the course of the detailed study of Simionatto et al. (1988) on thrombophlebitis following hematin administration (4 mg/kg), clotting times in nine healthy volunteers were monitored [156]. At the time of maximal plasma heme levels (~50 µg/mL), a prolongation of aPTT (+~24%), PT (+~20%) and TT (+~13%) was observed. The normal range was reached after 9 h (for aPTT), 24 h (for PTT), and 7 h (for TT) after hematin injection. For aPTT, the results were confirmed in vitro, when hematin (70 µg/mL) was added to untreated plasma (+~31%) [156]. Simultaneously, R. L. Jones (1986) hypothesized that in aged (up to 50 days, 10 mg/mL) hematin solution oxidatively degraded products are responsible for the anticoagulant effects [175]. In their approach, freshly prepared hematin solution did not cause any change of the clotting times (PT, TT, aPTT), whereas aged hematin solutions greatly prolonged the clotting times up to ~2.8-fold (aPTT, 50 days old solution, phosphate buffer, final concentration: 60 µg/mL) (Supplementary Table S2). Thus, the author determined the actual hematin concentration within the solutions by using the pyridine hemochromogen assay, revealing a 50% reduced actual hematin concentration. Indeed, chromatographic analysis (thin-layer and high-performance liquid chromatography) revealed a different pattern between aged and freshly prepared hematin solution. However, to date, structural elucidation and validation of these degradation products with anticoagulant function is missing. In order to prove the assumption of oxidative degradation products, R. L. Jones added antioxidant and iron-chelating compounds, which, indeed, were able to suppress the anticoagulant effect of the aged hematin solutions [175]. Furthermore, fast in vivo generation of the anticoagulant hematin degradation product(s) was suggested, which was derived from experiments in rats that received infusion of hematin (12 mg/kg) with a parallel monitoring of the plasma hematin concentration [175]. Finally, the author also compared the effect of the freshly prepared hematin solutions on the clotting times with that of Panhematin^®^. In contrast to freshly prepared hematin solutions, in case of Panhematin^®^ that was freshly reconstituted in water (as by instruction), a significant prolongation of the clotting times was recorded [175]. Due to these studies concerning the anticoagulant effect of aged hematin and Panhematin^®^ [157,175], Simionatto and colleagues spectroscopically examined the constitution of their hematin solution (Panhematin^®^). They detected changes in the absorbance spectra indicating the degradation of hematin. Thus, again, the anticoagulant effect was assigned to the potential degradation products of hematin by the authors [156].

In contrast to all other studies, Becker et al. (1985) observed a shortened partial thromboplastin time (PTT; by ~30%) induced by hematin (3 nmol) in vitro, which was interpreted by the authors as an hematin-mediated activation of the intrinsic blood coagulation cascade [176]. However, they also found an increase in fibrinolysis after hematin addition as determined by a pronounced decrease (by ~78%) of the euglobulin clot lysis time.

At the same time, clotting parameters in the presence of heme arginate were investigated in healthy volunteers (Supplementary Table S2) [158,177]. Tenhunen and coworkers injected heme arginate at a dose of 3 mg heme per kg and monitored coagulation parameters before and 15 to 240 min after injection. Consistent with the clinical trials (Section 2), no changes of the results obtained from the various hemostatic point-of-care tests (aPTT, PT (quick time), TT and ethanol gelation) were observed [158]. The same group repeated these experiments and correlated the results with the actual maximal plasma heme concentrations [177]. The latter was reached about 30 min after injection and determined to be ~51.5 µg/mL with a half-life of about 11 h. Again, the clotting times remained in the reference range and no significant changes were observed in the presence of heme (3 mg/kg) administered as heme arginate [177]. Unfortunately, the authors did not record these parameters at the time of the maximal plasma heme concentration. In 1989, Herrick et al. also monitored the hemostatic parameters of AIP patients that were treated with 3 mg/kg heme arginate. In most of the patients, heme arginate did not affect PT and aPTT. Only one out of 12 patients showed a prolonged aPTT, but upon both placebo and heme arginate treatment, suggesting an already preexisting coagulation disorder without any correlation with heme infusion [165].

Since 1990, further case reports of AIP patients, who were treated with Panhematin^®^, were published (Supplementary Table S2) [168,178]. For example, Gajra and colleagues intravenously infused 4 mg/kg of the hematin drug and monitored prolonged aPTT (~1.6-fold), FT (~1.2-fold), INR (from 1.19 to 1.52), and TT (~1.4-fold) 5 h after the treatment, which supported their clinical observations (Section 2) [168]. In contrast, Green and Ts’ao only observed a marginal increase of aPTT and PT after the second infusion of hematin, whereas the TT remained unaltered [178]. So far, there is no explanation for the differing results of both studies [168,178].

In 2003, Huang et al. tried a similar approach as Green et al. [174] using heme in complex with albumin and analyzing its effect on clotting times, but with the difference that Huang et al. aimed at the characterization of the heme-albumin complex as a new RBC substitute through its ability to carry oxygen [174,179]. Therefore, whole blood of rats was mixed with a recombinant human serum albumin (50 g/L)—heme (5 mM) solution (aqueous, 0.9% NaCl, pH 7.4). The analysis revealed no significant changes of the aPTT as well as of the PT [179].

Further projects followed that focused on the clarification of the effect of hemin on hemostatic parameters (Supplementary Table S2). The study of Rochefort et al. from 2007 supported the earlier results concerning a potential anticoagulant effect of heme with a high-frequency ultrasound technique [180]. The acoustic velocity of whole blood in rats, which underwent daily hemin treatment (50 mg/kg), was analyzed at 500 MHz. Upon hemin administration clotting velocity was significantly decreased up to a degree comparable to heparin treatment (500 IU/kg, daily). Whole blood from hemin-treated rats needed approximately 40 min to clot formation, while blood from control rats clotted much faster (within ~10 min). Furthermore, the effect was characterized by a decreased slope of the increase in acoustic velocity (0.005 m/s^2^ (hemin-treated) vs. 0.031 m/s^2^ (control)), and a lower final velocity (12 m/s (heme treated) vs. 30 m/s (control) after 100 min) [180]. The same group intraperitoneally injected pure hemin (50 mg/kg) in male Wistar rats, with hemin dissolved in 0.5% DMSO before administration. No significant change of the PT and aPTT was monitored here [181]. In the same year, Desbuards et al. described a preventive role of hemin for thrombosis. In the study, carotid thrombus formation in rats was induced by electrical stimulation [181]. The thrombus induced in all control rats accumulated RBCs and dystrophic endothelial cells, and was close to the necrotic intima layer. In contrast, only in two out of six hemin-treated rats thrombus formation was observed, but these thrombi were only consisting of a few cells and independent from the intima layer. In the other cases of hemin-treated rats, no thrombi were found [181]. In parallel, the authors examined tin-protoporphyrin IX (SnPPIX)-treated rats, which revealed the same extent and characteristics of thrombus formation as the control rats. Since SnPPIX is a highly potent inhibitor of HO-1 and hemin a potent inducer of the same, the authors suggested that the ability of hemin to prevent or minimize thrombus formation might be due to induction of HO-1 expression [181], which subsequently exerts vasoprotective actions [182,183]. These results were later confirmed by another group [184]. Their data also suggested that an enhanced HO-1 induction prevents thrombus formation. 30 mice (septic C57BL/5 model) were treated with 50 µmol/kg hemin before sepsis and, consequently, thrombus formation was induced by the cecal ligation puncture procedure (CLP) [184]. The authors calculated the number of thrombi in the liver, lungs and kidneys, while using HE and MSB staining. In control mice, CLP greatly increased the number of thrombi, while in hemin-treated mice the number of thrombi was significantly reduced: ~50% (liver, HE), ~35% (liver, MSB), ~67% (kidney, HE), ~50% (kidney, MSB), ~50% (lung, HE & MSB) [184]. Additionally, they again observed markedly prolonged PT and aPTT of freshly drawn plasma of the treated mice. Zinc protoporphyrin (ZnPPIX) as a known HO-1 inhibitor counteracted the effect of heme [184]. Later, Hassaan et al. confirmed these results in CLP mice [185]. Therefore, these studies revealed HO-1 induction as a possible explanation for the anticoagulant role of heme, which was proposed by the observation of prolonged clotting times. Moreover, the authors even suggest the therapeutic use of heme in patients with venous thrombosis [180].

De Souza et al. (2017) determined (via rotational thromboelastometry) shortened clotting and clot formation times ex vivo after the addition of heme (30 µM) to whole blood, suggesting in contrast to most of the other studies a procoagulant role of heme [186]. Moreover, just recently, hemin applied at concentrations of 1–100 µM was shown to have no significant effect on the aPTT of pooled plasma from healthy volunteers. The addition of human serum albumin (0.1%) did not change these results [187].

**Figure 2:**
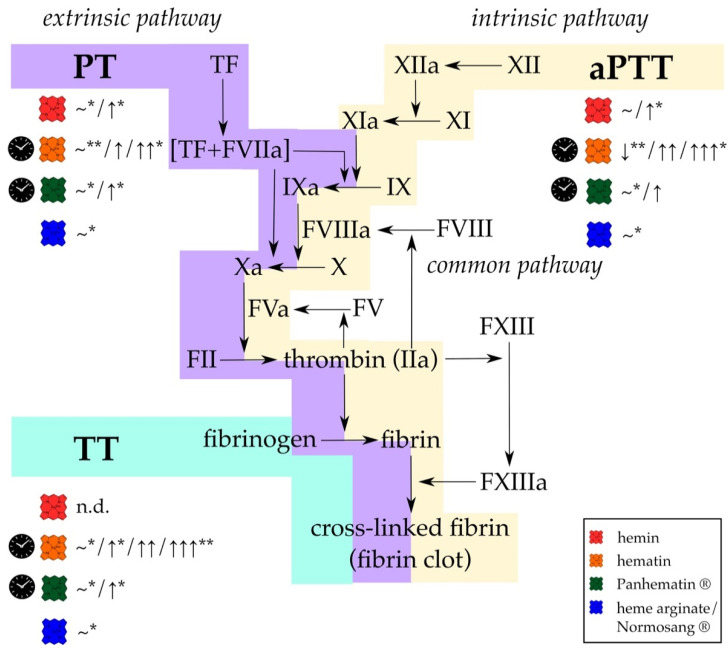
Different formulations of heme affect the results of standard coagulation diagnostic tests, (PT, aPTT, and TT) in vitro and in vivo. While PT is used to evaluate effects on the extrinsic and common pathway (violet), changes in the intrinsic and common pathways (yellow) can be determined using aPTT. With TT only the last step of fibrin generation and fibrin clot formation (turquoise) can be analyzed. Hemin (red symbol; concentration ranges: 50 mg/kg, 50 µmol/kg, 0.01–100 µM) either did not affect PT and aPTT or a slight prolongation was observed. TT was not determined (n.d.). In contrast, hematin (orange symbol; concentration ranges: 4–12 mg/kg, 3 nmol, 10–100 µg/mL) has been reported to induce significant prolongation of all clotting times. For Panhematin^®^ (green symbol; concentration ranges: 4 mg/kg, 70–78 µg/mL), longer clotting times were also recorded, but to a lesser extent than with hematin. However, from detailed investigations of different researcher it can be assumed that just aged hematin and Panhematin^®^ solutions can have such strong effects, whereas fresh hematin and Panhematin^®^ solutions are ineffective (clock symbol) [156,157,175]. Heme arginate/Normosang^®^ (blue symbol; concentration: 3 mg/kg) did not influence these diagnostic tests at all. FIIa = thrombin, FII = prothrombin, FV = factor V, FVa = activated factor V, FVIIa = activated factor VII, FVIII = factor VIII, FVIIIa = activated factor VIII, FIX = factor IX, FIXa = activated FIX, FX = factor X, FXa = activated FX, FXI = factor XI, FXIa = activated FXI, FXII = factor FXII, FXIIa = activated factor XII, FXIII = factor XIII, FXIIIa = activated factor XIII, n.d. = not determined, ~ = no effect, VWF = von Willebrand factor, ↓ = 30% decrease, ↑ = < 2-fold increase, ↑↑ > 2-fold increase, ↑↑↑ > 3-fold increase. * only observed in in vivo experiments; ** only observed in in vitro experiments.

Considering the reported changes of clotting times (in vivo and in vitro, Supplementary Table S2), the overall effect of pure hematin seems to be contradictory, describing either an anticoagulant, no or a procoagulant effect of heme on these hemostatic parameters (Figure 2). Panhematin^®^, a mixture of hematin and sorbitol, consistently showed a tendency to an anticoagulant impact, whereas Normosang^®^ (heme arginate) did not induce any changes of these hemostatic parameters (Supplementary Table S2; Figure 2).

In particular, the quality of the used hematin solutions should be considered. As reported, there might be a difference between the impact towards hemostatic parameters depending on whether a fresh or aged solution was infused [157]. In this context, fresh hematin solutions didn’t show any prolongation of clotting and bleeding times. Quite evident, degradative products of hematin were shown to cause the observed anticoagulant effects [156,157,175]. In addition, the experiments were performed in different laboratories by different researchers and also reagents, solvents, concentrations and measuring devices (Supplementary Table S2). Discrepancies between the studies with hematin were suggested to be due to batch-to-batch variations or patients’ diversity [178].

Altogether, it should be noted that between the first (1913) and the last (2020) report on the impact of heme (different formulations) on different clotting parameters there is a difference of more than 100 years (Supplementary Table S2). In total, presented studies of this section only involved 16 healthy test persons and 23 AIP patients. For that reason, there would be a need for more comparable studies in larger cohorts to unravel the actual effect of heme and its different formulations on clotting times as measured by standardized point-of-care test systems.

## 4. Heme Promotes Clotting Processes by Affecting Involved Cells

**Figure 3:**
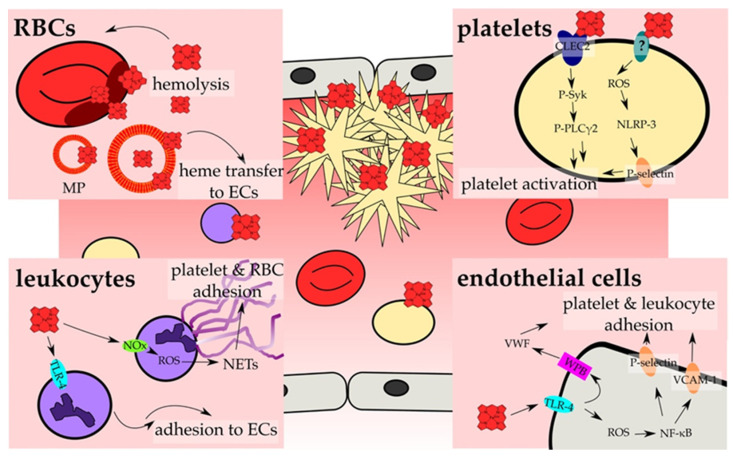
Heme activates cellular components of hemostasis. The main investigated pathways and consequences that result in the activation of cellular components of hemostasis and, thus, prothrombotic reactions by heme are depicted. On cellular level, hemostasis results from an interplay of RBCs (red, Section 4.3), platelets (yellow; Section 4.1), leukocytes (violet; Section 4.4) and endothelial cells (ECs, grey; Section 4.2). RBCs contribute to heme-induced hemostasis through the release of heme upon hemolysis, which can be further strengthened by heme itself (Section 4.3). Moreover, erythrocyte membrane particles (MP) incorporate and accumulate heme within the membrane, and allow for the transfer of heme to ECs (Section 4.2). These, in turn, become activated by heme in a TLR-4 -dependent manner (turquoise), which can lead either to the secretion of the contents of Weibel Palade bodies (WPBs; pink) (Section 4.2), among them VWF, or to ROS generation that triggers the increase of surface expression of adhesion proteins, such as P-selectin and VCAM-1 (orange) (Section 4.2). The exposure of those adhesion molecules as well as the secretion of VWF leads to the adhesion of platelets and leukocytes onto the endothelium. In contrast, activation of TLR-4 in leukocytes promotes the rolling and adhesion to ECs (Section 4.4). In addition, heme-induced NADPH oxidase (NOx; green)-dependent ROS generation in neutrophils can lead to NET formation, forming the scaffold for the adhesion of platelets and RBCs (Section 4.4). Finally, heme can also directly activate platelets. Two main mechanisms have been proposed. On the one hand, heme binding to CLEC2 was shown, leading to the phosphorylation of Syk (P-Syk) and PLCγ2 (P-PLCγ2) and eventually to the activation of platelets (Section 4.1). On the other hand, a ROS-dependent activation of the inflammasome via NLRP-3 has been demonstrated, which culminates in the expression of for example P-selectin, which again allows for the adhesion and activation of platelets (Section 4.1). Furthermore, the induction of ferroptosis (platelets) as well as apoptosis and necroptosis (endothelial cells) by heme has been demonstrated, which further might support the activation of endothelial cells and platelets (not shown; Section 4.1 and Section 4.2).

A variety of cell types, including platelets, endothelial cells, RBCs, and different kinds of leukocytes, is involved in the coagulation process [188]. Thus, it is not surprising that there are several studies which characterized the effect of heme on these cell types, linking heme and coagulation (Supplementary Table S3; Figure 3).

### 4.1. Heme and Platelets

Platelets are essential mediators of blood coagulation. Upon tissue injury, vasoconstriction leads to exposure of collagen, which can direct platelet adhesion, activation, and aggregation. A platelet plug is formed, and the wound is closed [189]. An increased number or a functional defect of these cells can be associated with an increased risk of thrombosis [4,53].

Alongside a broad study regarding the impact of hematin on different cell counts in rabbits, Brown microscopically determined the platelet count upon hematin injection [142]. Even small doses (no precise data given) of hematin instantly induced a loss of platelets that remained for several hours. Accordingly, with higher doses (no precise data given) the effect was greater (reduction up to >50%). For example, a single dose of 25 mg/kg hematin reduced the platelet count by approximately 70% 1 h after injection. When the treatment was stopped, the platelet count returned to normal state. The author assumed that the impact on the platelet count is due to the destruction of platelets by hematin, and might be an explanation for the observed hemorrhage (Section 2) [142]. About 70 years later, the same was observed in AIP patients that were treated with hematin [153,169]. 12 h after administration the platelet count was decreased by approximately 57% (from 227,000/mm^3^ to 98,000/mm^3^) [153]. In another AIP case report, the platelet count was decreased by ~12% (from 176,000/µL to 154,000/µL), 7 h after the fourth injection of hematin (4 mg/kg) [169]. These observations were further confirmed by in vivo studies (Supplementary Table S3) [169]. In a study by Glueck et al., blood samples were collected 10 min after infusion of 4 mg/kg hematin. The highest plasma heme level was then detected (4 mg/100 mL), and the platelet count was already decreased by approximately 41% [169]. Subsequent in vitro studies on the platelet aggregation of platelet-rich plasma revealed that hematin (0.1 mg/mL) triggers platelet aggregation. Neither preincubation of hematin with 1 mM adenosine nor 1 mg/mL apyrase could prevent from aggregation. When adenosine (70 µM), apyrase (0.07 mg/mL) or adenosine triphosphate (ATP; 2.5 mM) were added to the plasma sample prior to hematin treatment, hematin-induced platelet aggregation was inhibited [169]. When platelets were washed or gel-filtered they still aggregated upon hematin addition (1 µg/mL). Acetylsalicylic acid (0.12 mg/mL) counteracted this effect of hematin (2 µg/mL), but with a higher hematin concentration applied (5 µg/mL) platelet aggregation was again induced. Moreover, Glueck and coworkers observed a dose-dependent induction of ^14^C-serotonin secretion from platelets. For example, 0.1 mg/mL hematin induced a serotonin release of 10–12%, while 0.16 mg/mL hematin promoted a secretion of 82% [169]. ATP release by platelets was also increased by hematin. In conclusion, the authors suggested an hematin-induced platelet activation through the release of ADP [169]. Thus, the observed thrombocytopenia in patients correlates with the increased aggregation of platelets and not with a destructive effect of hematin (Supplementary Table S3). Additional treatment with anticoagulant agent heparin even worsened the coagulopathy in patients treated with hematin. Hence, the authors concluded that hematin has an anticoagulant effect [169].

In 1982, Peterson and coworkers suggested that epinephrine as a platelet agonist requires heme reduction in addition to binding the platelet α-adrenergic receptor to activate platelets [190]. Platelet aggregation was analyzed by using a dual-channel aggregometer, heme reduction by absorption determination at 558 nm (ε = 30 mM^−1^) [190]. Typical reducing agents (e.g., ascorbic acid) were shown to reduce heme, but did not affect platelet aggregation due to their incongruous structure for binding to the receptor. Only epinephrine, which possesses both properties, was able to induce platelet aggregation in vitro. The authors refer to heme that occurs in form of a heme-protein-complex in the membrane of platelets, near to the receptor. To further support their hypothesis, platelet aggregation induced by epinephrine was analyzed in the presence of heme-binding compounds (iron chelators, i.e., phenanthroline and dipyridyl agents). Indeed, these compounds inhibited the epinephrine-mediated platelet aggregation [190]. Later, Malik et al. (1983) confirmed these results, while demonstrating the ability of exogenously added heme to enhance ADP- and epinephrine-dependent platelet aggregation. In addition, binding of hemin to the platelet and the granule membrane was observed by ultrastructural localization via reaction of benzidine [191]. However, the underlying mechanism of heme-promoted platelet activation remained unresolved. Neely et al. (1984) aimed to decipher this mechanism with different approaches [192]. Again, hematin (2–5 µg/mL) was shown to promote aggregation of washed platelets. In comparison to the approach of Glueck et al. (1983), where hematin was added to platelet-rich plasma, a much lower (20- to 50-fold) hematin concentration was necessary to induce the same effect due to the absence of other plasma proteins that might scavenge hematin [169,192]. Microscopic ultrastructural analysis of the platelets confirmed the observed aggregation. The morphology of hematin-treated aggregated platelets was exactly the same as of platelets that were treated with typical platelet aggregation inducers (e.g., thrombin, collagen, ADP, and arachidonate). Moreover, hematin (5–10 µg/mL) triggered the production of thromboxane A_2_ (92.7–187.3 ng per billion platelets) in a dose-dependent manner, which was associated with the observed platelet aggregation. The incubation of platelets with acetylsalicylic acid prior to hematin treatment completely impeded thromboxane A_2_ generation [192]. The aggregation was independent of thromboxane A_2_ production, because with the addition of acetylsalicylic acid in the experimental setup hematin still induced platelet aggregation compared to the situation without acetylsalicylic acid. In contrast, hematin-induced platelet aggregation was inhibited by verapamil, apyrase, prostacyclin, and prostaglandin to different degrees. As all of these compounds abolish platelet aggregation through a downstream upregulation of cyclic AMP levels, Neely et al. suggested that cAMP levels might serve as a key player in hematin-induced platelet aggregation [192]. In addition, the effect of verapamil was counteracted by an increase of the calcium concentration, so that extra-platelet calcium might be of importance in hematin-induced platelet aggregation as well. The authors discussed that hematin can support the influx of calcium ions and other divalent cations into platelets. In a separate approach, alongside CaCl_2_, MgCl_2_, CoCl_2_, and SrCl_2_ increased hematin-mediated platelet aggregation, whereas mono- and trivalent cations did not show any effect. Other compounds, in particular thrombin and collagen, strengthened the aggregation effect of hematin in a synergistic manner. In two thirds of the donors, heparin potentiated hematin-induced aggregation, but only when induced with low hematin concentrations (around 1 µg/mL; precise range not given). Therefore, the authors recommended not to use heparin in parallel to hematin therapy [192].

Later, it was shown that in patients with myelodysplastic syndrome an improvement of cytopenia, such as an increase of the total platelet count, was determined upon treatment with heme arginate (Normosang^®^; 2–3 mg/kg) [162,193]. The same was observed in AIP patients that received heme arginate (3 mg/kg) and hematin (Panhematin^®^) infusion (4 mg/kg) [165,178]. As opposed to the aforementioned studies, Green and Ts’ao also found a lower platelet aggregation in this context [178]. Furthermore, the ATP and ADP content of the platelets was decreased, which allows for the conclusion of a potential degranulation of the platelets, a fact that was not recognized during electron microscopic analysis [178]. In contrast, in 2000 a decreased platelet count (from 129000/mm^3^ to 72000/mm^3^) was determined in an AIP patient after hematin (Panhematin^®^; 4 mg/kg) administration [168]. At about the same time, an albumin (50 g/L)-heme (3 mM) mixture was demonstrated to have no effect on the platelet count of a blood suspension from rats [179] as well as on the ADP-stimulated activation of platelets as demonstrated by the proportion of PAC-1 positive platelets in human whole blood [194].

In 2004, Peng et al. shed light on the role of elevated HO-1 expression in the prevention of platelet-dependent arterial thrombosis [195]. The authors observed that heme (15 mg/kg; intraperitoneal injection; twice daily) significantly accelerated platelet-rich thrombi formation in HO-1 knockout mice after stimulation with FeCl_3_, suggesting that hemin itself might have a prothrombotic effect. Moreover, the authors suggested a correlation of the heme-induced increase of oxidative stress and the observed accelerated thrombosis in HO-1 knockout mice, which cannot be prevented in the absence of HO-1. Upon hemin administration, platelet cGMP levels were significantly increased in wild-type and HO-1 knockout mice. However, when only hemin (40 mg/mL) was administered without pretreatment with FeCl_3_, no thrombotic phenomena were observed, neither in wild-type nor in HO-1 knockout mice. Therefore, the authors concluded that hemin itself might not affect the formation of platelet-dependent thrombi in their experimental setup [195].

After intraperitoneal injection of 50 mg/kg hemin in rats, Desbuards and colleagues determined a significant increase of the platelet count, but there was no sign of thrombus formation [181]. A dual role of hemin and hematin was suggested, exerting both anticoagulant and procoagulant functions. However, the potential relevance thereof is not yet understood. Heme might first promote platelet activation and second stimulate CO release through its degradation by HO-1, which further leads to the inhibition of platelet aggregation, e.g., through stimulation of soluble guanylate cyclase and upregulation of cGMP [181].

Since 2018, a ROS-dependent activation of platelets by heme was considered (Supplementary Table S3; Figure 3) [196]. In the course of their studies, Naveen Kumar et al. (2018) analyzed the cytotoxic effect of hemin on platelets, revealing a reduction of cell viability along with an elevated lactate dehydrogenase (LDH) release upon hemin treatment. Furthermore, platelet morphology was strongly affected. While lower concentrations (5–10 µM) provoked filopodia-like structures, higher concentrations (25 µM) led to the damage of the platelet membrane. Neither cytochrome c release nor caspase-3 activation was induced by heme, confirming a non-apoptotic cytotoxic effect [196]. Necroptosis was also disproved, since the necroptosis-specific inhibitor necrostatin-1 was not able to counteract heme-mediated death of platelets. However, when human platelets were treated with hemin (25 µM), an approximately six-fold increase of cytosolic ROS, depleted glutathione levels, and massive lipid peroxidation were detected. These effects were associated with an increased expression of HO-1 and subsequent elevated platelet iron levels. Thus, ferroptosis was suggested as a cause for heme-induced platelet cell death [196]. Furthermore, heme treatment of platelets was followed by elevated P-selectin levels, which is a marker for platelet activation, and the formation of platelet microparticles (PMPs). Interestingly, PMP generation was observed in several prothrombotic diseases, among these SCD [197]. The potent ferroptosis inhibitor ferrostatin-1 prevented all observed heme-mediated effects, observed in this study, suggesting that platelet activation and cell death are induced via ferroptotic pathways upon heme treatment [196]. In 2019, Naveen Kumar et al. analyzed these heme-mediated effects also in mice [198]. Thereby, a significant reduction of the total platelet number was observed. Moreover, for the first time it was shown that heme activated platelets through inflammasome activation in a NLRP-3 manner, driven by heme-induced ROS generation [198]. Interestingly, both heme-mediated ferroptosis and activation of platelets were prevented by melatonin, suggesting antioxidant melatonin as a potential drug for the treatment of thrombosis under hemolytic conditions [198].

Recently, Bourne et al. (2020) suggested that in contrast to endothelial cells (Section 4.2) platelets are activated by heme in a Toll-like receptor 4 (TLR-4) independent manner [199]. Instead, this process seems to be directed by an immunoreceptor-tyrosine-based activation motif receptor (ITAM receptor) based signaling pathway. Hemin at low concentrations (<25 µM) stimulates phosphorylation of the protein tyrosine kinase Syk and phospholipase C gamma 2 (PLCγ2). Indeed, addition of recombinant CLEC2 blocked platelet activation. Therefore, the authors concluded a crucial role of this receptor in heme-triggered platelet activation (Figure 3). Moreover, direct binding of heme to recombinant CLEC2 was demonstrated by applying spectroscopic methods, revealing a heme-binding affinity of ~200 nM. Heme-triggered aggregation of platelets was independent from oxidative stress, as the antioxidant N-acetyl cysteine could not prevent it. Interestingly, at higher hemin concentrations (>25 µM) platelet aggregation seemed to be independent from Syk. For that reason, the authors suggested that these high hemin levels might result in agglutination. Through the potency of recombinant CLEC2 in prevention of heme-driven platelet activation, it is suggested as a potential therapeutic agent against thrombosis in hemolytic patients [199].

### 4.2. Heme and Endothelial Cells

As the primary source of different molecules that participate in the clotting process, endothelial cells are pivotal for the regulation of blood coagulation. In healthy states, endothelial cells possess an anticoagulant and, in turn, antithrombotic nature through the secretion of various anticoagulants, such as thrombomodulin, EPCR or platelet inhibitors (e.g., NO, prostacyclin) [200]. Upon vessel injury, endothelial cells undergo activation which is followed by the expression of procoagulant proteins, such as TF and VWF. Subsequently, these proteins can initiate the coagulation cascade and trigger platelet activation, respectively [200]. Accordingly, in case of functional abnormalities the properties of endothelial cells may tend towards a prothrombotic nature [200].

In 1984, Neely et al. detected dose-dependent morphologic changes of bovine aortic endothelial cells (BAECs) after incubation with hematin (2–40 µg/mL, diluted in HEPES buffer) [201]. These alterations were reversible and marked by bulging, surface vesiculation, and cell retraction as determined with inverted phase-contrast light and scanning electron microscopy. When hematin was diluted in plasma, 40 µg/mL hematin caused the same extent of morphologic changes as 10 µg/mL hematin diluted in HEPES buffer. Thus, Neely and coworkers suggested that only unbound heme can affect endothelial cells [201]. In addition, the hematin-induced morphologic changes were attributed to hematin-triggered stimulation of contractile elements of the cells. These changes were also reversible, since the endothelial cells returned to their usual appearance after removal of hematin. While BAECs exposed to up to 40 µg/mL hematin did not show any sign of increased detachment, incubation with 100 µg/mL hematin resulted in a significant rise of cell detachment (from ~7.9% to ~13.0%). However, these effects of hematin might lead to an exposure of subendothelial structures with thrombotic consequences (Supplementary Table S3). This was further supported by an increased platelet adhesion to hematin-exposed BAECs [201]. Untreated BAEC monolayers bound less than 5% of platelets from a suspension of washed human platelets. When incubated with hematin (40 µg/mL) the platelet adhesion to endothelial cells was two times higher. Furthermore, addition of hematin to the platelet suspension increased adhesion even more. Via scanning electron microscopic examination, sites of adhesion were identified as matrix materials, plastic and the by hematin superficial roughened endothelial cells [201]. As a side effect, aggregates in the platelet suspension were observed after hematin addition, confirming the previously described results on the impact of hematin on platelets (Section 4.1). Finally, Neely et al. considered their observations as a potential basis for the clinically noticed thrombophlebitis and thrombocytopenia (Supplementary Table S3) [201].

Balla and coworkers observed alterations of porcine aortic endothelial cells (PAECs) upon exposure to hemin [202]. After fast heme uptake and accumulation within the plasma membranes, the cells were more sensitive towards oxidative stress as induced for example by H_2_O_2_, implicating a possible role in atherogenesis or hemorrhagic injury. This effect is mainly driven by heme-mediated LDL oxidation and was effectively prevented by the heme scavenger hemopexin [202,203].

In a study that aimed for the investigation of causes for iron-induced endothelial injuries as a consequence of hemolysis, Woollard and colleagues (2009) analyzed the effect of hemin for comparison [204]. Thereby, an isolated mouse aorta was perfused with 1 mM hemin resulting in a mild denudation of the endothelium, which was accompanied by collagen exposure and platelet aggregation [204]. A more extensive reaction of endothelial cells towards hemin exposure (10–50 µM) was demonstrated in 2012 [205]. Characterized by a concentration-dependent increase of LDH release, hemin induces apoptosis of BAECs. Only the administration of hemin with the highest applied concentration (50 µM) resulted in an increase of ATP. Moreover, apoptosis was accompanied by a significant appearance of cleaved caspase 3 and caspase 9 and, thus, is mediated via the mitochondrial intrinsic cell death pathway. Therefore, heme-induced mitochondrial damage was expected to occur prior to the initiation of endothelial cell death [205]. A heme-mediated decline of mitochondrial membrane potential and the suppression of basal respiration in BAECs further supported this assumption. Moreover, it was shown that heme-induced lipid peroxidation plays a major role in heme-mediated cell death. Here, hemin exposure of BAECs resulted in the formation of carbonyl adducts as well as thiol oxidation of proteins [205]. Interestingly, the level of LC3-II, the membrane-bound form of the central protein of autophagy LC3, is increased in BAECs 2 to 4 h after treatment with 25 µM hemin. In some cells also mitophagy was observed. Generated autophagosomes include lipid-protein adducts, suggesting a protective mechanism against hemin toxicity [205].

In 2014, Vercellotti and coworkers [206] infused heme (prepared as Panhematin^®^; 0.4–32 µmol/kg) into transgenic sickle mice (i.e., NY1DD) and wild-type mice leading to the development of vaso-occlusion in subcutaneous venules (19.7–38.9% stasis) only in sickle mice [206]. Pre-existing chronic hemolysis in sickle mice in comparison to wild-type mice might be the cause for the aforementioned observations, since heme-mediated vaso-occlusion clearly correlates with plasma heme levels. In fact, heme-induced stasis is associated with a high degree of adhesion molecules’ expression, such as of P-selectin and VWF. Heme-mediated initiation and potentiation of the expression of adhesion proteins (Section 3) was observed in vivo on vessel walls for both, sickle and wild-type mice. Moreover, in vitro heme exposure (20 µM) of HUVECs lead to the activation of the same with an increased expression of adhesion molecules on the cells’ surface, which might be dependent on NADPH oxidase and its activator protein kinase C (PKC) [206]. The authors identified TLR-4 signaling as the major trigger for vaso-occlusion in sickle cell mice, leading to NF-κB activation and, subsequently, expression of various adhesion molecules and degranulation of Weibel-Palade bodies [206] (Figure 3). However, as previously demonstrated for the heme-driven LDL oxidation in endothelial cells [202,203], also heme-mediated stasis was prevented by equimolar co-administration of hemopexin [206]. Furthermore, protoporphyrin (PPIX) antagonized heme-induced stasis, when intraperitoneally administered 1 h prior to heme.

Camus et al. (2015) realized that under the hemolytic conditions of SCD heme itself is transferred to the endothelium by circulating erythrocyte membrane microparticles (MPs), thereby promoting vaso-occlusion [207]. For the first time, it was demonstrated that a significant portion of cell-free heme (~one-third) binds to cell membrane fragments (Supplementary Table S3). In vitro, incubation of HUVECs with SCD-derived heme-laden MPs and synthetic heme-laden multilayer vesicles (MLVs) led to the incorporation of heme into HUVECs. In comparison to control erythrocyte MPs (contain ~20 nM heme), SCD erythrocyte MPs (contain ~65 nM heme) transferred heme in a 4-fold more efficient manner. Moreover, both SCD erythrocyte MPs and synthetic heme-laden MLVs triggered production of ROS and induced apoptosis, leading to strong endothelial toxicity and, thus, endothelial injury [207]. Since TLR-4 blocking resulted in a nearly complete inhibition of heme-laden MP-triggered ROS production, the authors concluded that the observed effects might be mediated in a TLR-4-dependent fashion, as already suggested by others for heme-mediated effects on endothelial cells [206] (Figure 3). In addition, hemopexin (2 µM) and the phosphatidylserine antagonist annexin-a5 (10 µg/mL) restrained heme transfer to HUVECs, ROS production and apoptosis. Hemopexin’s preventive impact was explained by its heme-scavenging properties. Furthermore, it seems to be capable of removing heme from MPs to a certain extent. The blockage by the phosphatidylserine antagonist prompted the authors to investigate a potential relevance of phosphatidylserine. Subsequent in silico studies suggested that MP-exposed phosphatidylserine binds heme with the help of two calcium ions. Further support of this prediction was gained by the fact that removal of calcium ions by complexation resulted in a reduced heme association to MPs [207]. However, in SAD mice (S-Antilles-D Punjab Hb-expressing SCD mouse model) heme-laden MPs induced kidney vaso-occlusions, while perfusion of 100 nM heme or MPs (300 MPs/µL) isolated from SAD mice was followed by a massive reduction of endothelial acetylcholine-dependent vasodilation in mouse mesenteric resistance arterioles, which was counteracted when MPs were preincubated with 1 µM hemopexin.

While Camus et al. (2015) observed heme-triggered apoptosis in HUVECs [207], in the setting of Singla et al. (2017) necroptotic pathways instead of apoptotic pathways were induced by heme [208]. Human lung microvascular endothelial cells (HLMVECs) showed a TLR-4-dependent loss of endothelial barrier stability upon hemin treatment (5–100 µM) as shown by a decrease of trans-endothelial electrical resistance along with an increase of monolayer permeability. These effects were abrogated by addition of a TLR-4-inhibitor, as well by the antioxidant N-acetylcystein and the iron chelator deferoxamine. Therefore, not only the activation of TLR-4-dependent pathways, but also heme-mediated ROS production might be important for these observations. In addition, heme’s iron moiety seems to be essential [208]. However, programmed cell death was triggered by hemin (40 µM) in a TLR-4- and ROS-dependent but caspase-3 independent manner. Necroptosis was confirmed by an increased activation of the mixed lineage kinase domain-like (MLKL) as a consequence of TLR-4 activation and ROS production [208] (Figure 3). The authors suggested that these molecular processes might contribute to the vaso-occlusive crises of SCD patients [208].

In context of atypical hemolytic uremic syndrome (aHUS), May et al. (2018) found different explanations for the procoagulant state of patients as well as for the complement activation on the level of the endothelium in vitro and in vivo (Supplementary Table S3) [209]. When macrovascular cells (i.e., HUVECs) were exposed to hemin (12.5–50 µM), increased HO-1 gene and protein expression as a mechanism of heme detoxification was observed. Moreover, the expression of the transmembrane receptor thrombomodulin was significantly upregulated. Due to its role in the anticoagulant protein C pathway, these changes might not only be relevant for heme-mediated regulation of the complement but also coagulation system [209]. However, these characteristics occurred to a lesser extent in microvascular endothelial cells (i.e., HMECs, GENCs, HRGECs). Thus, in particular the microvascular glomerular cell types (i.e., GENCs, HRGECs) were more susceptible towards heme-triggered complement activation, characterized by increased deposition of complement component 3 (C3). This is in good agreement with the commonly found microvascular thrombotic microangiopathic lesions in aHUS [209]. Overactivation of the complement system was also observed in mice, in particular in the kidney glomeruli, as a consequence of intraperitoneal hemin injection (40 µmol/kg). While thrombomodulin levels were elevated in skin and large liver vessels, the lung microvasculature showed decreased levels of thrombomodulin. Therefore, the authors suggested a procoagulant and complement-activating role of the microvascular endothelium under hemolytic conditions, which might be prevented by compounds with blocking properties towards heme toxicity [209]. Indeed, heme-induced (50 µM) elevated P-selectin expression on the surface of HUVECs as well as complement activation were prevented by hemopexin (5 µM), but not by HSA [210]. As Camus et al. (2015) already described [207], here again the relevance of RBC-derived MPs as heme carriers was demonstrated. Subsequent heme-dependent physiological consequences were again completely inhibited by hemopexin [210]. Heme-induced activation of endothelial cells was shown to not only be accompanied by an increased expression of adhesion proteins, like P-selectin and ICAM-1, but also by an elevated adhesion of HbS-RBCs to the endothelial cells, whose relevance was also demonstrated in SCD patients with vaso-occlusive crises [74].

### 4.3. Heme and RBCs

RBCs can participate in the blood coagulation process through provision of phosphatidylserine and microparticles, and subsequent initiation of thrombin generation [211]. Moreover, RBCs are incorporated into the thrombus through interaction with platelets and endothelial cells, which prevents from clot resolution. Therefore, quantitative and qualitative changes of RBCs, such as under hemolytic conditions, are accompanied by a higher incidence of thrombosis [32,211].

Microscopic studies of Brown (1913) revealed that the RBC count was affected by hematin administration to a much lesser extent in comparison to the platelet or leukocyte count [142]. However, while doses below 15 mg/kg hematin caused a decrease of the RBC count in rabbits with fast regeneration in only few cases, a dose of 20 mg/kg intravenously injected hematin directly induced a decline of the RBC count. Furthermore, Brown noticed that a daily injection of 10 mg/kg hematin was followed by a decrease of the RBC count by approximately 60% [142]. In addition, the RBCs were characterized by irregularities in size, variability of color, and the presence of immature cells, in particular basophilic cells. The reduction of the RBC count was assigned to hemolysis that was directly initiated by hematin itself. This assumption was supported by the observed hemoglobinemia [142]. In later published case reports of AIP patients, the injection of hematin was shown to induce a slight decline of the hematocrit without any obvious morphological changes of RBCs (Supplementary Table S3) [153,169]. At about the same time, other reports supported the hemolytic impact of heme on RBCs as kind of a feedback loop that might explain the decreased RBC count in the presence of high labile heme levels [212,213]. Heme (≥500 nM heme; lower concentrations not tested) accumulates in the RBC membrane, thereby inducing loss of intracellular potassium, which leads to hypotonic lysis of RBCs. Subsequently, swelling of RBCs was observed [212]. This was further accompanied by a decrease of glutathione, ATP, as well as hemoglobin levels. It was found that 5 µM heme was capable of inducing 50% hemolysis of RBCs after 2.5 h of incubation [212]. In another study, heme-induced RBC membrane instability and subsequent lysis was attributed to heme (20–500 µM)-triggered conformational alterations of cytoskeletal protein spectrin and protein 4.1 [214]. More recently, this was further supported by spectroscopic binding studies that aimed to characterize the heme-binding capacity of dimeric spectrin, revealing a K_D_ value of 0.57 µM [215]. Molecular docking suggested a specific binding site within the SH3 domain of erythroid spectrin [215].

Interestingly, the concentration of heme within the membrane of aged RBCs was demonstrated to be increased. Shaklai et al. (1985) proposed a potential role of heme in hemolysis and, thus, removal of aged RBCs from circulation [213]. Albumin was able to extract heme from RBCs membranes [213]. Moreover, heme-induced hemolysis (e.g., 50% hemolysis in case of 40 µM heme applied) was prevented in the presence of 1% of albumin, as a classical heme-scavenging protein.

In 2003, a mixture of albumin (50 g/L) and heme (3 mM) was shown to not induce any change of the RBC count, which again demonstrated the protective role of albumin towards heme toxicity [179]. Noticeably, the same was observed when hemin (50 mg/kg) was injected daily in normal Wistar rats—neither hematocrit nor the RBC count was significantly changed (Supplementary Table S3) [181].

### 4.4. Heme and Leukocytes

Leukocytes play a role in coagulation through the production of cytokines that can modulate the expression of pro- and anticoagulant proteins as well as adhesion molecules [216,217]. In addition, they can directly interact with other vascular cells, including platelets and endothelial cells. Dysregulation of leukocyte activation or abundance may thus lead to thrombotic complications [217]. In particular, monocytes and neutrophils contribute to a procoagulant state, in particular through an elevated expression of the procoagulant TF on their surfaces (Section 5.8) [218].

In 1911, W. H. Brown reported a large number of polymorphonuclear leukocytes after hematin and hemin injection in rabbits, as determined at sites of accumulation of the pigments by microscopic analysis [138]. A few years later, the author extended these experiments and directly compared the effect of hematin intoxication with those of the control injections [142]. Leukocytosis (e.g., ~1.4-fold total leukocyte count) was, thus, observed in rabbits characterized by an increase of large (e.g., ~5.6-fold) and small (e.g., ~1.5-fold) mononuclear leucocytes. In addition, there was a decrease of eosinophiles and an increase of basophiles. Cells were counted 11 days after a single injection of 10 mg/kg hematin. Four days after a daily dose of 20 mg/kg hematin, a marked increase of large mononuclear leukocytes (~10-fold) and a higher number of polymorphonuclear cells (~7.8-fold) was detected. The total number of leukocytes was 4.6-fold increased. Not exclusively, but partially these effects were attributed to the alkaline solution [142]. Similarly, in patients with myelodysplastic syndromes a great rise of neutrophils after treatment with heme arginate (Normosang^®^; 2–3 mg/kg) was observed [162,193]. However, a direct connection of heme-neutrophil interaction with coagulation was proposed by Smith and Winslow (1992), when they identified heme as a stimulating agent of the procoagulant activity of isolated human peripheral blood mononuclear cells (PBMCs) [219]. Furthermore, they suggested that the procoagulant effect of stroma-free hemoglobin solutions might be also due to heme itself [219].

Considerably later, it was shown that ^111^In-labeled leukocytes quickly migrated and accumulated in different organs of mice, such as liver and spleen, after intravenous infusion of hemin (intravascular concentration: 750 µM) [220]. In parallel, lesions were registered supporting the role of heme as an inflammatory mediator. Interestingly, Arruda and coworkers demonstrated a significant delay of neutrophil apoptosis by heme (1–50 µM) in vitro, which is inevitably associated with the de novo synthesis of antiapoptotic proteins (e.g., Bcl-x_L_, interleukin (IL)-8) [221,222]. A particular role of the Ras/Raf/MAPK and phosphoinositide 3 kinase (PI3K) pathways for the heme-mediated protection of neutrophils was suggested, since inhibitors of these pathways completely reversed the heme effect. Heme itself promoted extracellular signal-regulated kinase (ERK)-2 translocation to the neutrophil nucleus and triggered PKC-dependent protein kinase B (Akt) phosphorylation, a key step in PI3K/Akt signaling, and, thus, the degradation of proapoptotic proteins (e.g., Bad) [221,222]. In the presence of SnPPIX, an inhibitor of heme oxygenase, the protective effects of heme were partially revoked. The authors suggested an important role of heme degradation products in the mediation of neutrophil survival. However, neither biliverdin nor bilirubin was able to protect neutrophils from apoptosis [221]. In addition, ROS production and consequently redox potential changes of the cells might be crucial for the heme-mediated antiapoptotic effect on neutrophils, very likely through NF-κB activation by heme [221,222]. In the presence of albumin, neither a difference of the chemotactic effects of heme on neutrophils [222] nor a change of the total leukocyte number in blood suspensions from rats (Supplementary Table S3) [179] were observed, displaying the protective role of albumin towards heme’s effects on leukocytes.

At the same time, Wagener et al. (2003) confirmed the accumulation of large amounts of heme (no precise data available) at sites of injuries in a wound-healing model in rats [223]. As a consequence, an enormous infiltration of leukocytes occurred, mainly consisting of granulocytes (after 1 day) and macrophages (after 3 days) [223]. The number of lymphocytes did not change. These observations were confirmed when 750 µM hemin was intradermally injected in rat mucosa [223]. There is evidence that the leukocyte influx is triggered by inflammatory, chemokine-dependent pathways, as for example granulocytes exposed to heme exhibited increased IL-8 expression [222]. In order to unravel the role of heme oxygenase (HO) in heme-induced leukocyte recruitment, the mucosa was pre-exposed (24 h) to the HO inhibitor tin mesoporphyrin (20 µM) prior to hemin treatment [223]. An even more aggravated number of granulocytes and macrophages infiltrated into the mucosa, confirming again the protective role of HO [223]. Desbuards et al. (2007) also observed a higher number of leukocytes in rats, after they were treated either with 50 mg/kg hemin per day or with a mixture of hemin (50 mg/kg per day) and the HO-1 inhibitor SnPPIX (60 mg/kg per day) for a period of seven days [181]. While other effects of hemin were attributed to an induction of HO-1 and consequent prevention of thrombosis, these data are not in accordance with this hypothesis but fit well to the observations of Wagener et al. (2003) (Supplementary Table S3) [181,223].

A few years later, Belcher et al. (2014) noticed increased rolling and subsequent adhesion of leukocytes to the endothelium in subcutaneous venules of sickle mice (NY1DD) after infusion of heme (3.2 µmol/kg; prepared as Panhematin^®^) [206]. This occurred in a TLR-4-dependent manner, thus, supporting the hypothesis raised by Arruda et al. (2004) that already suggested a NF-κB-dependent signaling as the underlying mechanism for heme-induced effects on leukocytes [206,221]. At the same time, Chen et al. (2014) identified heme as an inducer of neutrophil extracellular traps (NETs) generation in vitro and in vivo [224]. NETs were shown to be capable of capturing RBCs and platelets as well as to be part of venous thrombi [225,226], hence why these findings are of great importance to understand the role of heme as a procoagulant molecule. In SCD mice, the authors observed NET formation in pulmonary blood vessels, which were increased when pre-stimulated with the cytokine TNFα. The same was observed in TNFα-treated hemizygous mice after intraperitoneal hemin injection (50 µmol/kg). TNFα-primed wild-type bone marrow neutrophils were stimulated with 10–20 µM hemin and produced NETs in a dose-dependent manner (Figure 3). Neither PPIX nor ZnPPIX induced the formation of NETs in primed neutrophils. As already described elsewhere for the heme-mediated activation of neutrophils [130,222], NET formation occurred as a consequence of intracellular upstream of ROS by heme (Figure 3). Since neither heme nor TNFα alone induced NET formation, the authors suggested that a predisposal of neutrophils by proinflammatory cytokines is necessary to become sensitive for heme-mediated NET generation [224]. Hemopexin was able to abrogate the observed NET generation by scavenging heme, whereas addition of HSA only slightly reversed the effect [224]. In 2017, heme-induced NET formation was fluorometrically quantified demonstrating a dose-dependency in the presence of 1.5–15.3 µM hemin [227]. In human neutrophils, nuclear swelling and slight structural NET-like changes were observed upon hemin treatment (7.7–15.3 µM). The authors confirmed that heme-mediated NET production was ROS-dependent, more precisely NADPH oxidase-derived ROS-dependent. In contrast to the observations of Belcher et al. (2014) concerning neutrophil adhesion [206], NET formation was not TLR-4-dependent (Supplementary Table S3) [227].

## 5. Direct Influence of Heme on Coagulation Proteins

**Figure 4:**
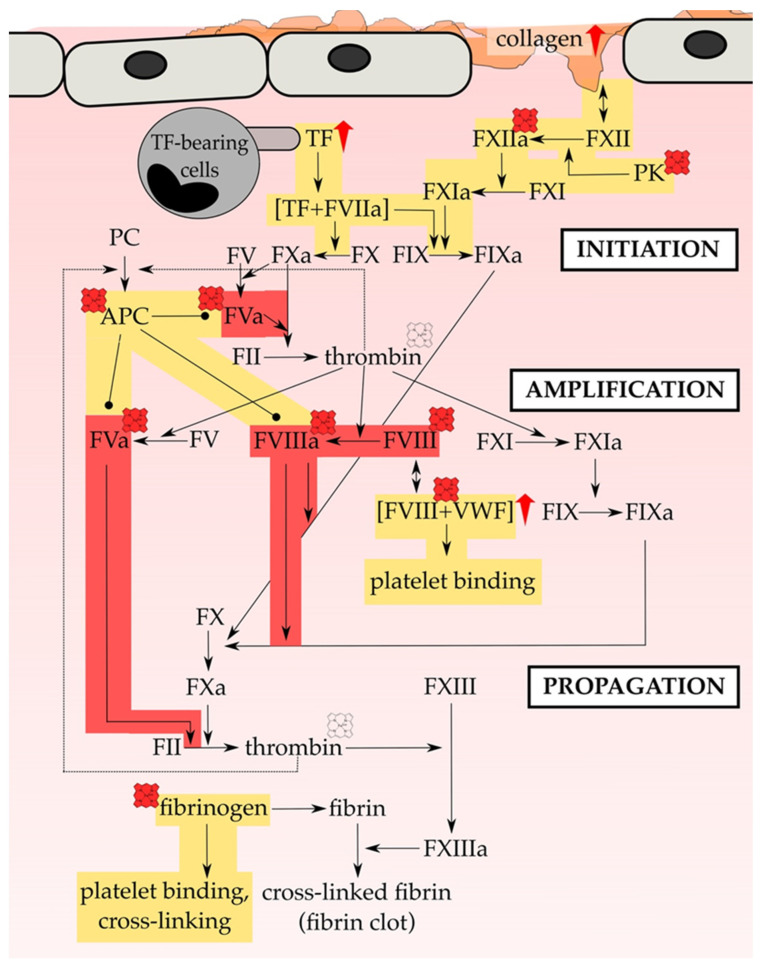
Heme promotes plasmatic hemostasis. Heme can directly affect various proteins of the blood coagulation cascade. While usually activated through the exposure of TF (e.g., by monocytes or the subendothelium), in pathologic thrombotic situations hemostasis activation through the exposure of negatively charged surfaces (e.g., of collagen) plays a supportive role. Indeed, initiation, amplification and propagation of hemostasis on the surface of cells (TF-bearing cells and platelets) is targeted by heme, either through upregulation of proteins’ expression level (red arrow) or regulation of proteins’ function (heme symbol). Direct heme-binding with functional consequences was only demonstrated for APC, FVIII(a) and fibrinogen. Contradictory results were obtained in case of the impact of heme on the activity of thrombin (pale heme symbol). The investigations of more than 35 years research allow for the assumption that heme is able to initiate hemostasis via both the upregulation of TF expression on leukocytes and endothelial cells as well as of collagen in the subendothelium. Most of the analyzed interactions tend to a procoagulant/prothrombotic (yellow) impact of heme. In contrast, heme-induced FVIIIa and FVa inactivation is exclusively described leading to anticoagulant (red) consequences. FVIII and FV are central cofactors of the coagulation cascade. Thus, the inactivation of FVIIIa and FVa by heme could constitute kind of a control center of heme-mediated initiation, amplification and propagation of the coagulation process. Plasma level changes of clotting factors that were recorded in humans upon heme infusion are not included. APC = activated protein C, FIIa = thrombin, FII = prothrombin, FV = factor V, FVa = activated factor V, FVIIa = activated factor VII, FVIII = factor VIII, FVIIIa = activated factor VIII, FIX = factor IX, FIXa = activated FIX, FX = factor X, FXa = activated FX, FXI = factor XI, FXIa = activated FXI, FXII = factor FXII, FXIIa = activated factor XII, FXIII = factor XIII, FXIIIa = activated factor XIII, PC = protein C, PK = plasma kallikrein, VWF = von Willebrand factor.

Early signs of heme as an actor in blood coagulation with partially controversial results and unresolved questions led to the assumption that there must be other relationships apart from the effects on hemostatic cellular components [142,153]. Hence, researchers started to consider a direct interference of heme with the clotting cascade [169]. In this context, heme-mediated regulation of protein expression levels or transient heme binding to proteins with functional consequences were suggested [97,169,178,228]. This is applicable to several procoagulant (e.g., TF and fibrinogen) and anticoagulant (e.g., activated protein C (APC)) proteins, as described in the following (Supplementary Table S4, Figure 4).

### 5.1. Heme Interaction with Fibrin(ogen)

In the course of the development of porphyrins as photosensitizing agents for photodynamic therapy, W. H. Howell observed in 1921 that hematoporphyrin IX prepared from hemin induced a change of fibrinogen’s solubility [229]. As a consequence, thrombin was unable to convert fibrinogen in its coagulable form fibrin [229]. Later, this was confirmed by others, who suggested direct binding of hematoporphyrin to fibrinogen [230,231]. Musser et al. (1979) supported this assumption by chromatographic techniques [232]. In contrast, no binding to fibrin, neither in its crosslinked (with factor XIIIa) nor in its non-crosslinked form, was observed [233].

In 1983, Glueck et al. demonstrated that intravenous hematin (4 mg/kg) infusion in an AIP patient resulted in a decline of fibrinogen level (from ~2.8 mg/mL to ~1.9 mg/mL) and a rise of FDP (~2-fold), 10 min after infusion [169]. Complete recovery was reached 48 h after infusion. Moreover, the authors showed that fibrin polymerization was not affected by hematin upon preincubation of fibrin monomers with hematin (20–90 µg/mL) [169].

Based on the first results indicating a potential interaction of hematin with fibrinogen, Green and colleagues analyzed direct binding by size-exclusion chromatography (SEC) with a sephadex G-200 column [174]. Co-elution of fibrinogen and hematin was observed, which was interpretated as hematin binding to fibrinogen. Within this study, hematin was solved in 0.25% sodium carbonate solution for 24 h prior to usage. Due to other studies that followed shortly after, the formation of anticoagulant acting heme degradation products was assumed (Section 3) [156,157,175]. At about the same time, the same group demonstrated that upon a 30 min preincubation with hematin (stored at 4 °C in sodium carbonate buffer) fibrinogen binding to gel-filtered platelets was induced [192]. Again, hematin was not used in a fresh state, but, in contrast to the previous reports, here, hematin acted in a procoagulant manner.

In line with the unaltered clotting times in the presence of heme arginate (Section 3, Figure 2), 3 mg/kg heme arginate (Normosang^®^) did not cause any change of fibrinogen, FDP, and fibrinopeptide A plasma levels in healthy test persons [177].

More than 20 years later, Nielsen et al. (2011) suggested at least one permanently bound heme group in fibrinogen [234]. This hypothesis was only based on the finding of the [M + H]^+^ ion of heme after LC-MS/MS analysis of fibrinogen and needs further evidence. Moreover, addition of nitric oxide/hydroquinone prevented from CORM-2-mediated procoagulant effects, thus suggesting heme-mediated carbon monoxide sensing by fibrinogen [234]. The presence of one or more permanent heme-binding sites in fibrinogen, however, has not yet been proven so far.

At the same time, Barrera et al. (2011) suggested fibrinogen as a hemozoin-binding protein [235]. Exposure of hemozoin (isolated from *Plasmodium falciparum* cultures) with plasma resulted in an enrichment of host fibrinogen in hemozoin. Binding of one molecule fibrinogen to approximately 25,000 hemozoin-heme molecules was estimated from SDS-PAGE [235]. Further experiments are required to confirm and characterize the proposed direct interaction and the ratio between the interaction partners. In human monocytes, the putative hemozoin-fibrinogen complex caused TLR-4-mediated oxidative stress. Furthermore, binding of different cell types, such as platelets and endothelial cells, via fibrinogen receptors might play a role in malaria pathogenesis [235].

In 2013, K. Orino immobilized fibrinogen on Sepharose 4B beads and demonstrated binding of hemin (10 µM) to bound fibrinogen spectroscopically [236]. Moreover, the author observed that fibrinogen-bound hemin still exerted peroxidase-like activity, which was comparable to unbound heme [236].

Direct heme binding of fibrinogen was further confirmed by Ke and Huang (2016), who demonstrated a shift of the Soret band of hematin (20 µM) to 410 nm upon complexation by fibrinogen [237]. Raman spectroscopy allowed for the assumption of a hexacoordinated complex. Functional studies under non-thermal plasma exposure revealed that hematin (50–500 µM)-treated blood and plasma showed rapid superficial clot layer formation. This layer consisted of cross-linked protein polymers with high molecular weight (>245 kDa). The same was observed with pure fibrinogen (10 mg/mL) solution, suggesting that hematin (30 µM) induces cross-linking of fibrinogen (Figure 4). Since there are no free thiol-groups in fibrinogen, the authors excluded cross-linking by disulfide bonding, but suggested heme-triggered dityrosine formation [237]. This was confirmed by fluorescence spectroscopy and ultra-performance liquid chromatography as well as by specific chemical modification of tyrosine residues, which completely abolished hematin-triggered cross-linking of fibrinogen [237]. Hou et al. (2018) confirmed these results of heme-induced fibrinogen cross-linking [238]. Again, direct hemin binding to fibrinogen was characterized by a red-shifted Soret band and a K_D_ of ~3.3 µM [238]. Additionally, a conformational change of fibrinogen (100 nM) with an introduction of an α-helical structure by hemin (25 µM) was observed by circular dichroism spectroscopy. One binding site within the γ-chain around the residues R^256^, F^293^, T^371^, K^373^, T^374^ and Y^377^ was suggested by applying an automatic docking tool [238]. Hemin (25 µM) binding to fibrinogen (50 nM) also increased heme’s peroxidase-like activity (ΔAbs ~0.17). Thus, the authors suggested the usage of this interaction for the label-free detection of fibrinogen in plasma samples [238].

In agreement with the fact that heme is capable of inducing cross-linking of fibrinogen by itself, just recently it was shown that heme (0.01–50 µM) does not have any impact on the amidolytic activity of the fibrin stabilizing active factor XIII (400 nM) [187].

Several researchers started to analyze the properties of the fibrinogen-heme complex. Therefore, there is no longer any doubt that heme binds to fibrinogen. However, the interaction remains not fully characterized. For instance, the binding affinity of heme towards fibrinogen or the actual heme-binding site(s) are still not known.

### 5.2. Heme Interaction with Factor VIIIa (FVIII(a)) and VWF

In 1967, Davis et al. discovered an inhibitory effect of hemin-derived hematoporphyrin on FVIII, which was attributed to a hematoporphyrin-driven destruction of FVIII [239].

About 15 years later, Glueck and colleagues observed reduced FVIII level upon infusion of 4 mg/kg hematin in an AIP patient [169]. Determined by a two-stage chromogenic assay [169], it can also indicate a reduced activity of FVIIIa instead of a decline of plasma concentration. Indeed, Green et al. (1983) demonstrated a concentration-dependent decrease of FVIII procoagulant activity in plasma in the presence of hematin (24 h aged; 18–70 µg/mL). The activity was reduced by up to 72% and 88% in pooled plasma and commercial FVIII concentrate, respectively [174].

These studies laid the foundation for an analysis of a potential direct interaction of hematin with FVIII. In 1986, Green et al. showed co-elution of the FVIII/VWF complex (36 U/mL of vWF:Ag) with hematin (0.17 mg/mL) after 30 min preincubation in SEC (Sepharose CL-4B column), suggesting binding of hematin to the procoagulant complex [240]. Indeed, the dissociation of FVIII from VWF was inhibited by hematin and binding of the FVIII/VWF-hematin complex to platelets was observed. The authors hypothesized that previous activation of platelets by hematin is required [240].

25 year later, apart from direct interactions, hemin (10 µM) was also reported to induce the formation of VWF fibers (ultra-large VWF) on the surface of endothelial cells as a consequence of Weibel Palade body (WPB) exocytosis [228]. VWF secretion by endothelial cells was later also observed by others, showing that it occurs in a TLR-4-dependent manner [26,206] (Figure 3). Due to the important role of VWF in platelet recruitment and clot formation this might be a crucial step in heme-triggered prothrombotic events.

Approximately at the same time, direct binding of heme in form of hemin was confirmed by UV/vis spectroscopy, revealing a Soret band shift to ~412 nm as well as rather high heme-binding affinities for the full-length (Helixate^®^; K_D_ ~12.7 nM) and the B-domain-deleted (ReFacto^®^; K_D_ ~1.9 nM) version of recombinant FVIII (rFVIII) [241]. Furthermore, a total number of ~10 heme-binding sites with heterogeneous affinity was estimated. The procoagulant activity of full-length (up to 50%) and B-domain-deleted (up to 51%) FVIII was impaired by heme (5 min preincubation) in a dose-dependent manner, as determined by routine factor X generation assay. In contrast to earlier reports [174], hematoporphyrin showed no impact on FVIII’s procoagulant activity [241]. The procoagulant activity of FVIII is caused by its cofactor properties towards factor IXa and thus, the ability to support the generation of active factor X. Repessé and colleagues demonstrated that the interaction of rFVIII with FIX is impaired in the presence of heme (100-fold molar excess; by ~52%), thereby explaining the inhibition of FVIII’s procoagulant activity [241]. In contrast, the interaction of FVIII with VWF, platelets and phosphatidyl serine was not altered. Interestingly, thrombin was still able to cleave FVIII when heme was present. VWF, but not albumin (up to 200-fold molar excess), protected FVIII from heme-driven inactivation. The authors thus concluded that VWF hides heme-binding sites rather than scavenging the heme [241].

So far, it is unclear whether or not the reported effects of heme on FVIII are relevant for in vivo situations. Although there is a massive release of labile heme under hemolytic conditions, it remains unclear whether consequently secreted VWF prevents from the anticoagulant effect that was observed in case of FVIII in vitro. As one of the most important cofactors within the blood coagulation cascade, the inactivation of FVIII by heme could also play an important role as a central point for the control of heme-triggered prothrombotic effects (Figure 4).

### 5.3. Heme Interaction with Factor V (FV)

Glueck et al. (1983) recorded declined FV levels upon intravenous infusion of hematin (4 mg/kg) in an AIP patient [169]. Moreover, hematin was capable of reducing the activity of FV (by ~80%), as detected 10 min after hematin infusion (Supplementary Table S4; Figure 4) [169]. However, this is the only report on the effect of heme on FV to date and, thus, many details of a potential direct interaction are missing.

### 5.4. Heme Interaction with Factor XII (FXII)

After the observation of a decreased PTT (Section 3; Supplementary Table S2), Becker et al. (1985) searched for a potential effect of different porphyrins, including hematin, on the FXII-dependent pathway [176]. A dose-dependent increase of the amidolytic activity of the serine protease kallikrein towards a fluorogenic peptidic substrate (Bz-Pro-Phe-Arg-*p*-nitroanilide) was observed (e.g., in the presence of 12 nmol hematin, ~10-fold faster conversion), when human plasma was 10 min preincubated with hematin (3–24 nmol). When plasma was deficient of FXII, prekallikrein (inactive precursor of kallikrein) or high-molecular-weight kininogen (cofactor for kallikrein and FXII activation), hematin was unable to trigger the conversion of the fluorogenic substrate by kallikrein. Addition of a specific FXII inhibitor (CHFI) and the serine protease inhibitor SBTI completely abolished the hematin-mediated activation of kallikrein. Due to interference of hematin within the spectrophotometric measurements, the effect on the amidolytic activity of FXII towards a chromogenic substrate could not be analyzed. However, the authors demonstrated that PPIX was capable of increasing FXII amidolytic activity (78.6 µg/mL FXII, 38 µM PPIX) and autoactivation (9.7 µg/mL FXII, 1.9 µM hematin) [176]. The results clearly suggest a procoagulant effect of hematin on the FXII-dependent pathway of blood coagulation (Figure 4). Therefore, controversy to earlier reported anticoagulant effects of hematin were attributed to different preparations of hematin, since within this study hematin was freshly prepared in alkaline solution as the main difference to earlier studies [176]. However, blocking of the intrinsic, FXII-dependent pathway in vivo did not influence coagulation activation by heme (35 µmol/kg) in mice, which demonstrated that it is only dependent on the extrinsic, TF-driven pathway of coagulation [242] (Section 5.7). These results might refute the relevance of the in vitro results from Becker et al. (1985). Though, it is unclear to what extent heme would still bind to components of the FXII-dependent pathway in vivo under hemolytic conditions, while supporting its procoagulant effect on the extrinsic pathway of coagulation (Figure 4).

### 5.5. Heme Interaction with Thrombin

When Glueck et al. (1983) preincubated (0–180 min) thrombin (25 U) with hematin (70 µg/mL), clotting time was incubation time- and temperature-dependently prolonged, which led the authors to conclude a direct impact of hematin on thrombin [169]. A few years later, the generation of fibrinopeptide A resulting from the reaction of thrombin (0.25 U/mL) and fibrinogen (2.5 mg/mL) was radioimmunologically analyzed in the presence of four weeks aged hematin (6 µg/mL). Thrombin was preincubated (15 min) with hematin. As a consequence, fibrinopeptide A generation was ~100-fold blocked [243], which fits well to the investigations of Glueck et al. on thrombin and FDP in the presence of hematin [169] (Section 5.1). This also suggests that the resulting anticoagulant effect might be due to degraded hematin and not by hematin itself, as already shown for prolonged clotting times after hematin infusion [156,175] (Section 3). The same group suggested the possibility of direct binding of hematin to thrombin, after both co-eluted in SEC as also observed for fibrinogen (Section 5.1) [174]. Moreover, hematin was able to inhibit the amidolytic activity of human α-thrombin towards a fluorogenic peptide substrate (D-Phe-Pro-Arg-5-aminoisophthalic acid dimethyl ester) by up to 86.3% (Supplementary Table S4) [174]. Once again, a 24 h old hematin solution was used, which might have influenced the results (as demonstrated later [156,157,175]).

More than 30 years later, Sparkenbaugh et al. (2015) detected elevated thrombin-antithrombin (TAT) level (up to 2.5-fold) upon retro-orbital injection of 35 µmol/kg heme in mice, which is a sign for coagulation activation [242]. In a time-dependent experiment, coagulation activation occurred 1 h after heme injection and was still increased 6 h after injection [242]. The authors demonstrated that this was not due to a direct interaction of heme with thrombin but caused by heme-induced TF expression (Section 5.7) [242].

In contrast to the studies of Green et al. from 1983 [174], hemin (0–50 µM) was recently shown to not influence the amidolytic activity of human α-thrombin (25 nM) [187] (Figure 4). While in the latest study a chromogenic peptide substrate (p-Glu-Pro-Arg-MNA) was used, in 1983 a fluorogenic substrate was added. Therefore, the earlier registered inhibitory effect could be caused by heme-associated fluorescence quenching and the results might be affected. However, the differences in heme preparation, buffer systems, and used substrates could also explain the different results.

### 5.6. Heme Interaction with Plasmin(Ogen)

For the first time in 1983, the effect of hematin on fibrinolysis was evaluated [174]. 15 µg/mL hematin (24 h kept in sodium carbonate buffer) was capable of inhibiting whole blood clot lysis. When hematin was first mixed with plasmin (0.017 U/mL) and then applied to the clot, the same result was obtained suggesting a direct, inhibitory effect of hematin on plasmin. Therefore, the impact of hematin (0.006–0.09 µg/mL) on the amidolytic activity of plasmin was analyzed. The ability of plasmin to cleave a fluorogenic peptide substrate (D-valine-leucine-lysine-5-aminoisophthalic acid dimethyl ester) was inhibited by 6–50% [174]. This was the very first report on a hematin-protein interaction that proposed a procoagulant effect of hematin. However, at the same time, plasminogen levels were shown not to be changed after infusion of hematin (4 mg/kg) in an AIP patient [169]. As already hypothesized for fibrinogen (Section 5.1) [234], also for plasmin and its inhibitor α2-antiplasmin a putative heme group was identified via LC-MS/MS, but has not yet been further investigated [244].

### 5.7. Heme Interaction with Adhesion Proteins

Apart from VWF, other adhesion proteins have also been reported to be upregulated by heme. For instance, already in 1997, Wagener and colleagues observed increased superficial expression of intercellular adhesion molecule 1 (ICAM-1; two-fold), vascular cell adhesion molecule 1 (VCAM-1; 3-fold), and E-selectin (4-fold) upon incubation of HUVECs with heme (50–100 µM, for 24 h) by using different techniques (e.g., dot blot immunoassay, ELISA) [245]. Later on, these observations were confirmed in mice that received a dose of 750 µM heme by intravenous infusion [220]. In particular, the surface of vascular endothelial cells in liver and pancreas were affected. While the expression of ICAM-1 and VCAM-1 was still increased after 24 h, P-selectin expression was only elevated 1 h after administration [220].

In 2009, Woollard et al. demonstrated that hemin even slightly induced endothelial collagen expression as demonstrated after perfusion of 1 mM hemin in mice aorta [204]. This might allow for the heme-triggered initiation of the intrinsic contact pathway of blood coagulation (Figure 4).

Moreover, hemin (10 µM) exposure of HUVECs resulted not only in an increase of WPB exocytosis but also of P-selectin expression on the surface of the cells within 5 min [228]. However, inhibition of TLR-4 completely blocked this effect, suggesting heme-induced P-selectin expression as a TLR-4 dependent process [228]. Then, Frimat et al. (2013) then confirmed the parallel expression of P-selectin on the surface of HUVECs and the exposure of VWF [26]. Due to the rapidity of expression, the authors suggested a correlation with WPB mobilization [26]. Later, these observations were also made by others in vitro and in vivo (Supplementary Table S4) [206].

These results are of great importance, since these adhesion proteins enable the recruitment of different cell types to the endothelium. However, the molecular basis of the proposed interactions is still not entirely solved. Within the process of blood coagulation there are even more adhesion molecules involved, like the platelet endothelial cell adhesion molecule 1 (PECAM-1), but a regulation by heme was not yet explored. Thus, further investigation for a complete understanding of the role of adhesion proteins in heme-triggered coagulation is required.

### 5.8. Upregulation of TF by Heme

After several studies that reported the endothelial activation and adhesion molecule expression by heme, Setty et al. were the first who suggested a direct effect of heme on TF [246]. With different techniques, i.e., ELISA and flow cytometry, the authors found that heme (1–100 µM) dose-dependently increases TF expression (up to 50-fold) on the surface of micro- (HLMECs) and macrovascular (HUVECs) endothelial cells. Moreover, heme (100 µM) upregulated TF mRNA expression (up to ~17-fold) after incubation with endothelial cells. TF mRNA expression occurred slower and to a lower degree than cytokine-induced (e.g., by TNFα) [246]. Indeed, NF-κB activation inhibitors (i.e., sulfasalazine, curcumin) prevented from heme-triggered TF mRNA expression, suggesting a dependency of heme-induced TF mRNA expression from NF-κB activation. In line with a TF mRNA expression upregulation, total endothelial protein levels of functionally active TF were raised up to 20–39-fold after 4–7 h, tending towards a strong procoagulant effect [246].

A few years later Rehani et al. (2013) made the same observations in PBMCs and monocytes. 4 h incubation with 10 µM hemin yielded a ~40-fold and ~70-fold increase of TF activity, as determined by a one-stage clotting assay [247]. Hemopexin (15 µM) attenuated this effect. TF mRNA levels were also increased in monocytes (140–350-fold) after incubation with heme (2–4 h). Efficient impact of inhibitors led to the conclusion that heme might trigger TF expression via TLR-4, PKC, NADPH oxidase, and ERK-1/2 [247], as already shown for the expression of adhesion molecules on the surface of HUVECs [206] (Section 4.2).

In support of these studies, Souza and colleagues (2014) investigated heme-driven TF expression in PBMCs and neutrophils by applying different techniques (i.e., thromboelastometry, thrombin generation test) [248]. Heme (30 µM)-induced TF expression in PBMCs was confirmed, whereas no TF expression could be observed in neutrophils. In addition, the hypercoagulable state was characterized by a decreased coagulation time and time to maximal velocity [248].

Evidence for in vivo regulation of TF regulation by heme was provided by Sparkenbaugh and colleagues in 2015, who investigated TF-dependent coagulation activation in mice [242]. Indeed, inhibition of TF by an antibody abolished heme (35 µmol/kg)-driven coagulation activation in mice, as detected by TAT plasma levels. Subsequently, the authors demonstrated that 6 h incubation with heme (5–50 µM) induced TF expression as well increased TF activity in human PBMCs and RAW 264.7 mouse macrophages in combination with a dose-dependent increase of procoagulant activity. In contrast, heme did not induce TF expression in endothelial cells [242]. Further in vivo studies demonstrated that knock-out of TF in myeloid cells, hematopoietic cells or endothelial cells still allowed for heme-triggered coagulation activation. In mice with human TF expressed on hematopoietic cells and murine TF on non-hematopoietic cells, the authors observed that blocking of only both TF sources abolished heme-mediated coagulation activation. However, also in SCD mice elevated TAT level were recorded, which might confirm the previous observations that heme activates the coagulation cascade via the TF-dependent pathway. Again, these effects were completely prevented by hemopexin administration (280 µmol/kg) [242].

After De Souza and colleagues observed shortened clotting times upon addition of 30 µM heme to whole blood (Section 3), a TF-specific antibody was used to block its activity. As a consequence, heme-induced coagulation was inhibited, thus, again providing evidence for the relevance of TF in heme’s procoagulant role [186] (Figure 4).

The molecular basis for this interaction has not been entirely explored. Just recently, first insights were provided by a preprint from Hounkpe et al. (2020) [249]. Herein, heme (≥10 µM)-induced TF expression in PBMCs was shown as well as a dose-dependent (5–30 µM heme) increase of TF activity. Moreover, plasma mixed with heme (30 µM) for 4 h exhibit increased TF procoagulant activity, as detected by a one-stage clotting assay. Indeed, blocking of TLR-4 resulted in a complete inhibition of heme-triggered TF procoagulant activity. Thus, also this procoagulant effect of heme seems to be mediated through TLR-4 [249]. A second, very similar receptor for heme was suggested by others [250]. May et al. (2020) demonstrated direct heme binding (~2–3:1 heme:receptor; K_D_ ~6.78 µM) to the receptor for advanced glycation end products (RAGE), which resulted in receptor oligomerization [250]. Thereby, signaling of heme via RAGE was followed by the phosphorylation of ERK-1/2 and Akt, as also earlier shown by others [221,222,247]. Experiments in wild-type and RAGE knockout mice suggest an involvement of RAGE in heme-triggered TF expression, since TF expression was less in RAGE knockout mice [250].

However, more investigation is required to unravel the whole network of heme-induced signaling pathways that lead for example to elevated TF expression, and, thus, to prothrombotic states.

### 5.9. Heme Interaction with Anticoagulant Proteins

To date, the effect of heme on the anticoagulant pathways is largely unknown. Antithrombin-III levels were shown to be influenced neither by the injection of hematin (4 mg/kg) in AIP patients [169] nor by the infusion of 3 mg/kg heme arginate (Normosang^®^) in healthy volunteers [177]. In contrast, in CLP mice administration of hemin (50 µmol/kg) clearly resulted in an upregulation of protein C and APC plasma levels as well as in a decrease of thrombomodulin plasma levels [184]. These effects that tend to an anticoagulant action were attributed to the role of hemin as an inducer of HO-1 [184].

In 2020, transient heme binding to APC was demonstrated for the first time [187]. This binding was characterized by a K_D_ of ~400 nM. Moreover, experimental studies allowed for the assumption of two heme-binding sites that subsequently were identified within the serine protease domain (heavy chain) of the enzyme (i.e., T^285^GWGYHSSR^293^, W^387^IHGHIRDK^395^) with Y^289^ and H^391^ serving as the coordinating residues [187]. A molecular dynamics simulation suggested a conformational change upon binding of two heme molecules, as seen by a more rigid light chain of the protein. Furthermore, in complex with heme (0.01–50 µM), plasma-derived APC (50 nM) was inhibited in its amidolytic activity with an IC_50_ of ~10.41 µM and a K_i_ of ~12.56 µM. For the recombinant APC (Drotrecogin alfa, Xigris ^®^) an inhibition by heme (IC_50_ of ~3.88 µM) was also observed [187]. As already observed for the fibrinogen-heme complex (Section 5.1; [238]), the APC-heme complex exhibits an increased peroxidase-like activity (~512.33% in comparison to 100% by heme only), as well. Moreover, heme (≥ 10 µM) abolished the anticoagulant activity of APC (5 nM) as demonstrated with an aPTT-based clotting assay, displaying a procoagulant role of heme. Indeed, human serum albumin (0.1%) could protect APC up to a certain extent. However, under these conditions, again, 100 µM heme was able to inhibit the anticoagulant activity of APC. Interestingly, the cytoprotective function of APC was not affected by heme. Quite the contrary, APC (20 nM) was capable of preventing heme (120 µM)-induced hyperpermeability of HUVECs, and, thus, protecting from heme-driven cytotoxicity [187]. Thus, this was the first detailed report on a direct interaction of heme with one of the endogenous clotting inhibitors (Supplementary Table S4). Although this effect of heme has not been proven in vivo so far, the interaction of heme with APC seems to be versatile. Beyond the formation of a peroxidase-like complex, and direct protection of endothelial cells from heme-directed loss of permeability by APC, heme inhibits the anticoagulant function of APC, which results in the support of procoagulant reactions.

Beside direct upregulation of procoagulant factor and the promotion of their procoagulant activities, heme, thus, is also capable of the inhibition of an inhibitor of the blood coagulation cascade, that usually targets FVa and FVIIIa (Supplementary Table S4, Figure 4).

## 6. Conclusions

This review presents labile heme as a multifaceted molecule with versatile effects in the context of blood coagulation. Extensive studies over the past ~110 years described the consequences of the infusion of heme and its different formulations in in vivo studies and case reports, as well as the impact of heme on different cell types and proteins that participate in the coagulation process.

Originally, Pierach and Rosborough suggested both an anti- and a procoagulant role of heme [251]. In early studies, heme (as hemin or hematin) was infused in rather high concentrations (10–180 mg/kg; single exception: ≥3.5 mg/kg) into animals resulting in symptoms of bleeding, i.e., hemorrhage, ecchymosis, (internal) bleeding, and hematomas (Figure 1). In contrast, in humans, healthy volunteers or AIP patients, lower amounts of heme (1.2–6 mg/kg) were administered (as hematin or heme arginate), which commonly caused thrombotic complications, including thrombophlebitis and bile thrombi (Figure 1). Opposed to these observations, but in line with the animal studies, heme (primarily used in the form of hematin) caused prolonged aPTT, PT, and TT (Figure 2). To date, however, it is unclear to which extent the anticoagulant symptoms were caused by heme itself. Several studies demonstrated that long-storage of heme in solution might lead to the generation of oxidative degradation products that are responsible for the observed effects [156,157,175]. In more recent studies, freshly prepared heme had either no or a shortening effect towards clotting times, suggesting a rather procoagulant role.

Accordingly, several cell types participating in blood coagulation, including platelets, RBCs, endothelial cells, and leukocytes, were shown to be affected by heme in a prothrombotic manner (Figure 3). Heme-induced hemolysis of RBCs as a feedback mechanism was not yet directly considered in the context of heme-triggered prothrombotic consequences. This process might lead to a potentiation of the observed effects due to the release of even more labile heme into the vascular compartment. Through heme-induced conformational changes of RBC membrane proteins the RBC membrane stability can be reduced. Furthermore, heme can accumulate in RBCs’ membrane. Thus, RBC membrane microparticles are able to transfer heme to the endothelium, which can, in turn, activate endothelial cells. Indeed, heme infusion was associated with direct effects on the endothelium, such as the ACH-dependent vasodilation. Activation of TLR-4 by heme triggers the exocytosis of WPBs, which results for example in the release of VWF [26,206,228] (Figure 3). Furthermore, the expression of adhesion proteins is upregulated by heme in a TLR-4 and ROS-dependent fashion [206,208]. The exposure of VWF and different adhesion proteins, such as P-selectin and VCAM-1, enables the adhesion of platelets and leukocytes to the endothelium, promoting procoagulant processes. As the heme-driven endothelial activation, also the prothrombotic processing of leukocytes is mediated via TLR-4. Activation of TLR-4 by heme again triggers the adhesion of leukocytes to the endothelium [206]. In addition, neutrophils might respond with the formation of NETs upon heme exposure, which is mediated via the NADPH oxidase and consequent ROS generation [227] (Figure 3). Again, adhesion of platelets and RBCs is facilitated. Apart from these signaling pathways, also necroptotic and apoptotic pathways may play a role in heme-triggered endothelium activation [208,209].

Heme-triggered denudation of endothelial cells along with an exposure of collagen has been shown to be necessary for heme-associated platelet adhesion and consequent aggregation. Overall, two main mechanisms have been described for the platelet activation by heme. Direct heme binding to C-type lectin-like receptor 2 (CLEC2) induces a tyrosine kinase network that leads finally to the activation of platelets [199]. In addition, heme-induced ROS generation can promote the expression of adhesion proteins, that, in turn, enable the adhesion and subsequent activation of platelets [196,198]. In contrast to endothelial cells, for platelets, the relevance of ferroptotic signaling has been shown [198].

Leukocyte accumulation in various organs, such as the liver and spleen, was observed as a consequence of heme exposure [220]. As already described for the endothelium activation by heme, heme-induced leukocyte rolling and adhesion also occurred TLR-4-dependently [206]. Moreover, heme-triggered neutrophil migration and recruitment were accompanied by PKC activation, oxidative stress, and actin polymerization [221,222]. However, heme-induced NET formation might be an essential step, since NETs are not only able to promote proinflammatory signaling but can also stabilize blood clots [224,252].

Finally, within the last few years, a remarkable development towards the investigation of heme as a mediator of protein expression and activity could be observed (Figure 4). In particular, the procoagulant proteins TF, fibrinogen, and VWF seem to be essential key targets that confer heme a procoagulant character (Figure 4). The inhibition of the anticoagulant activity of APC clearly supports this assumption. In contrast, the interaction of heme with FVIII(a) tends to an anticoagulant character of heme. This can be prevented by VWF, suggesting an inferior role of the proposed interaction or a counteracting effect together with the interaction of heme on FV. Since FV and FVIII are the central cofactors within the blood coagulation cascade, their interaction with heme might serve as a control mechanism that prevents from further potentiation of thrombosis at a certain state.

A potential interference of heme with several other proteins of the coagulation process, such as factor IX or factor X, was not yet examined. Furthermore, heme-binding affinities are only known for fibrinogen, FVIII(a) and APC. In the future, the characterization of the heme-binding capacity of other clotting factors and/or inhibitors might complete the whole picture of the range of the role of heme as an effector molecule in the blood coagulation cascade. However, due to its interference with different components at various steps of the coagulation system, it is already obvious that heme cannot only trigger coagulation activation, in particular by effects on endothelial cells and platelets as well as by the upregulation of relevant factors (e.g., TF and VWF), but can also promote further clotting through initiation, amplification, and propagation of plasmatic hemostasis.

This review provides a comprehensive overview of the broad range of heme’s actions as a modulator of blood coagulation, thereby in particular emphasizing its relevance in hemolysis-driven thrombosis. A role of heme in the development of thrombotic complications in SCD has in particular been proposed [18,253].

Furthermore, the detailed insights summarized herein regarding the molecular basis of heme-triggered coagulation allow for the targeted investigation of the missing interrelations and as yet unknown potentially regulatory heme–protein interactions. Finally, the actual role of heme in the coagulation system and as a promoting factor for prothrombotic events in hemolytic patients depends on the availability and quantity of heme and plasmatic proteins, including clotting factors, anticoagulant/fibrinolytic proteins and adhesion proteins, the heme-binding affinity of those proteins which directly bind heme, the location of relevant cell types with respective target receptors, such as TLR-4, and the chronology of events. An in-depth analysis of these factors might help to entirely understand the molecular basis of heme-triggered thrombosis in hemolytic disorders and support the development of suitable therapies within the future. Overall, due to the conspicuous clinical manifestation of thrombophilic reactions upon hemolysis, heme should be considered in the treatment of these complications, rather than focusing on a sole treatment of thrombotic symptoms [17,20].

## Data Availability

Not applicable.

## Supplementary Materials

The following are available online at https://www.mdpi.com/2077-0383/10/3/427/s1, Table S1: Overview of side effects observed after heme injection, Table S2: Overview of heme effects on bleeding and clotting times, Table S3: Overview of the impact of heme intoxication on cells participating in blood coagulation, Table S4: Overview of the impact of heme and its formulations on proteins acting in blood coagulation.
