# Supplementary material for: Linking Labile Heme with Thrombosis
Source: J Clin Med. 2021 Jan 22;10(3):427. doi: 10.3390/jcm10030427 (PMC7865584; doi:10.3390/jcm10030427)
Supplement: Supplementary file 1 [file jcm-10-00427-s001.pdf]

Journal of
Clinical Medicine

Supplementary Materials

Linking labile heme with thrombosis

Marie-Thérèse Hopp [1] and Diana Imhof [1,*]

[1] Pharmaceutical Biochemistry and Bioanalytics, University of Bonn, An der Immenburg 4, 53121 Bonn, Germany; mhopp@uni-bonn.de (M.H.); dimhof@uni-bonn.de (D.I.)
* Correspondence: dimhof@uni-bonn.de

Received: date; Accepted: date; Published: date

Supplementary tables

Supplementary table S1. Overview of side effects observed after heme injection.

Observed side effect	Organism	Compound	Solvent	Dose	Injection	Effect	Reference
Ecchymosis, hemorrhage	Guinea pig, rabbit	Hematin, hemin	0.85% salt solution	30 - 54 mg/kg	intraperitoneal, subcutaneous, intravenous	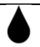	Brown 1911 [1]
Hemorrhage	Cat, dog	Hematin	0.85% NaCl + 2% Na_2CO_3 alkaline solution	3.5 - 9 mg/kg	intraperitoneal, visceral	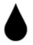	Brown & Loevenhart 1913 [2]
Hemorrhagic kidney injury, hemorrhage, anemia	Rabbit	Hematin	0.85% NaCl + 2% Na_2CO_3 alkaline solution	25 mg/kg	intravenous	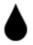	Brown 1913 [3]
Hemorrhage, hyaline thrombi, emboli, vaso-occlusion, vascular injury, infarcts	Rabbit	Hematin	0.85% NaCl + 2% Na_2CO_3 alkaline solution	10 - 25 mg/kg	intravenous	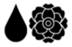	Brown 1913 [4]
Hemorrhages, thromboses, infarctions	Dog	Hematin	disodium buffer, pH 7.6	N/A (intravenous), 200 mg (intraperitoneal, 3 x weekly), 20 mg	intravenous, intraperitoneal, subcutaneous	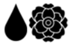	Anderson et al. 1942 [5]

				(subcutaneous, 5 x in a period of 1-2 weeks)			
Inhibition of clotting mechanism	Dog	Hematin	physiological saline, sodium salt solution	14.5 - 32.6 mg/kg	intravenous		Corcoran & Page 1945 [6]
Massive hemorrhagic bleeding, bleeding out of every body orifice	Rat	Hematin	isotonic Na_2CO_3 solution	100 - 180 mg/kg	intravenous		Gessler et al. 1966 [7]
Thrombophlebitis	Human (hepatic porphyria patients)	Hematin	0.25% Na_2CO_3 , pH 8.0; diluted in physiologic saline	1.2 - 6 mg/kg (applied as 4.3 - 108 mg/l, daily)	intravenous		Dhar et al. 1975 [8]
Internal bleeding, bleeding into small intestine and petechiae of liver, lungs, adrenals, hemorrhage, subcutaneous hematomas	Rat	Hemin	1% Na_2CO_3	40 - 60 mg/kg	intravenous		Lips et al. 1978 [9]
Chemical phlebitis	Human (AIP patients)	Hematin	saline solution	1.8 - 3.7 mg/kg (applied as 2 mg/ml, daily)	intravenous		Lamon et al. 1979 [10]
Thrombophlebitis	Human (AIP patient)	Hematin	N/A	196 mg, every 12 hours	intravenous		Morris et al. 1981 [11]
Phlebitis	Human (AIP patients)	Hematin	1% Na_2CO_3	4 mg/kg (every 12 hours or daily)	intravenous		McColl et al. 1981 [12]
Bile thrombi	Human (Protoporphyria patients)	Hematin	0.25% Na_2CO_3, pH 8.0; diluted in physiologic saline	3 - 4 mg/kg	intravenous		Bloomer & Pierach 1982 [13]
Occasional thrombophlebitis	Human (AIP and variegate porphyria patients)	Heme arginate (Normosang®)	N/A	2 - 3 mg/kg (applied as 25 mg/ml, repetitive)	intravenous		Mustajoki et al. 1986 [14]

Occasional bleedings	Human (myelodysplastic syndrome patients)	Heme arginate (Normosang®)	0.9% saline solution	2 - 3 mg/kg (applied as 25 mg/ml, repetitive)	intravenous	💧	Ruutu et al. 1987 [15]
Thrombophlebitis	Rabbit	Hematin (Panhematin®)	aqueous solution with 40% 1,2-propanediol and 10% ethanol	5 mg/kg (applied as 25 mg/ml, repetitive)	intravenous	❁	Tenhunen et al. 1987 [16]
Thrombophlebitis, fibrotic events, vaso-occlusion	Human (healthy)	Hematin (Panhematin®)	0.05 M Na_2CO_3, 0.04 M sorbitol	4 mg/kg	intravenous	❁	Simionatto et al. 1988 [17]
Phlebitis	Human (AIP patients)	Heme arginate (Normosang®)	0.9% NaCl	3 mg/kg	intravenous	❁	Herrick et al. 1989 [18]
Occasional thrombophlebitis	Human (AIP patients)	Heme arginate (Normosang®)	physiological saline	3 mg/kg (Finland), 250 mg (France)	intravenous	❁	Mustajoki et al. 1993 [19]
Coagulopathy, hematoma	Human (AIP patient)	Hematin (Panhematin®)	0.05 M Na_2CO_3, 0.04 M sorbitol	4 mg/kg	intravenous	💧	Gajra et al. 2000 [20]
Thrombotic complications	Human (acute porphyria patients)	Hematin (Panhematin®)	0.05 M Na_2CO_3, 0.04 M sorbitol	< 2 - > 6 mg/kg	intravenous	❁	Anderson & Collins 2006 [21]
Thrombosis	Human (acute porphyria patients)	Heme arginate (Normosang®)	N/A	3 mg/kg (applied as 25 mg/ml, repetitive)	intravenous	❁	Marsden et al. 2015 [22]

💧 = Evidence for a tendency to an anticoagulant effect of heme and its formulations. ❁ = Evidence for a tendency to a procoagulant effect of heme and its formulations.

Supplementary table S2. Overview of heme effects on bleeding and clotting times.

Parameter	Organism	Compound	Solvent	Dose	Injection	Setting	Baseline [s]	Monitored time [s]	Change	Effect	Reference
**aPTT**	Human (AIP patient)	Hematin	N/A	196 mg, every 12 hours	intravenous	Case report	25-41	> 60[a]	> x1.5	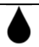	Morris et al. 1981 [11]
aPTT	Human (AIP patient)	Hematin	N/A	4 mg/kg, every 12 hours	intravenous	Case report	37.5	>150[b]	> x4.0	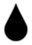	Glueck et al. 1983 [23]
aPTT	Human (AIP patient)	Hematin	N/A	4 mg/kg	intravenous	*in vivo*	~30	~100[c]	~ x3.3	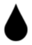	Glueck et al. 1983 [23]
(a)PTT	Human	Hematin	0.1 N NaOH, diluted in PBS	3 nmol	- (plasma sample)	*in vitro*	461	323	~30% ↓	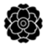	Becker et al. 1985 [24]
aPTT	Human	Hematin	1 N NaOH, diluted in potassium phosphat buffer (pH 7.5)	60 µg/ml	- (plasma sample)	*in vitro*	37.4 (0 h) - 35.8 (50 d)	40.9 (0 h) - 103.1 (50 d)	≤ x2.9 (aged hematin exclusively)	-/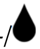	Jones 1986 [25]
aPTT	Human	Hematin	1 N sodium carbonate (pH 7.5))	60 µg/ml	- (plasma sample)	*in vitro*	37.4 (0 h) - 35.8 (50 d)	39.8 (0 h) - 183.0 (50 d)	≤ x4.6 (aged hematin exclusively)	-/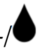	Jones 1986 [25]
aPTT	Human	Hematin (Panhematin®)	water	78 µg/ml	- (plasma sample)	*in vitro*	N/A	N/A	x1.4	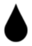	Jones 1986 [25]
aPTT	Rat	Hematin	1 N sodium carbonate (pH 7.5))	12 mg/kg	intravenous	*in vivo*	17.3	N/A	~ x1.8 (3 min after injection)	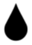	Jones 1986 [25]

aPTT	Human	Heme arginate (Normosang®)	aqueous solution + 40% 1,2-propanediol + 10% ethanol	3 mg/kg	intravenous	*in vivo*	~34.5	~34.3[d,e]	No significant change	-	Tenhunen et al. 1987 [16]
aPTT	Human	Heme arginate (Normosang®)	physiological saline	3 mg/kg	intravenous	*in vivo*	~35.0	~33.0 - 36.0[f]	No significant change	-	Volin et al. 1988 [26]
aPTT	Human	Hematin (Panhematin®)	0.05 M Na_2CO_3, 0.04 M sorbitol	4 mg/kg	intravenous	*in vivo*	N/A	N/A	~ x1.2	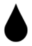	Simionatto et al. 1988 [17]
aPTT	Human	Hematin (Panhematin®)	0.05 M Na_2CO_3, 0.04 M sorbitol	70 μg/ml	- (plasma sample)	*in vitro*	N/A	N/A	~ x1.3	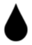	Simionatto et al. 1988 [17]
aPTT	Human (AIP patients)	Heme arginate (Normosang®)	0.9% NaCl	3 mg/kg	intravenous	*in vivo*	N/A	N/A	No significant change	-	Herrick et al. 1989 [18]
aPTT	Human (AIP patient)	Hematin (Panhematin®)	0.05 M Na_2CO_3, 0.04 M sorbitol	4 mg/kg	intravenous	Case report	27	28 (1 hour after 1st injection), 33 (1 hour after 2nd injection, day 4)	If, only very slight change after 2nd injection	-	Green & Ts'ao 1990 [27]
aPTT	Human (AIP patient)	Hematin (Panhematin®)	0.05 M Na_2CO_3, 0.04 M sorbitol	4 mg/kg	intravenous	Case report	35.6	58.3	~ x1.6	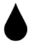	Gajra et al. 2000 [20]
aPTT	Rat	Albumin-heme (50 g/l & 3 mM, respectively)	0.9% NaCl, pH 7.4	10 - 44%	- (whole blood sample)	*in vitro*	~30.0	~30.0	No significant change	-	Huang et al. 2003 [28]

aPTT	Rat	Hemin	< 0.5% DMSO	50 mg/kg	intraperitoneal	*in vivo*	~16.9	~17.8	No significant change	-	Desbuards et al. 2007 [29]
aPTT	Mouse (CLP)	Hemin	DMSO, diluted in PBS	50 µmol/kg	intraperitoneal	*in vivo*	~19.0	~22.0	~ x1.2	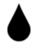	Fei et al. 2012 [30]
aPTT	Mouse (CLP)	Hemin	DMSO, diluted in PBS	50 µmol/kg	intraperitoneal	*in vivo*	~9.2	~15.6	~ x1.7	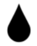	Hassaan et al. 2015 [31]
aPTT	Human	Hemin	0.03 M NaOH, diluted in DPBS	1 - 100 µM	- (plasma sample)	*in vitro*	~31.3	~36.7 (1 µM) – 41.4 (100 µM)	No significant change	-	Hopp et al. 2020 [32]
aPTT	Human	Hemin + albumin (0.1%)	0.03 M NaOH, diluted in DPBS	1 - 100 µM	- (plasma sample)	*in vitro*	~32.0	~32.9 (1 µM) - 33.7 (100 µM)	No significant change	-	Hopp et al. 2020 [32]
**Bleeding time (Duke)**	Rabbit	Hematin	0.85% NaCl + 2% Na_2CO_3 alkaline solution	25 mg/kg	intravenous	*in vivo*	n.d.	> 7200	n.d.	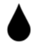	Brown 1913 [3]
**Clotting time**	Rat	Hemin	< 0.05% DMSO	50 mg/kg	intraperitoneal	*in vivo*	~600	~2400	~ x4.0	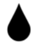	Rochefort et al. 2007 [33]
Clotting time	Human	Hemin	0.1 M NaOH, adjusted to pH 7.4	30 µM	- (whole blood)	*ex vivo*	~500	~400	~20% ↓	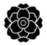	De Souza et al. 2017 [34]
**Clot formation time**	Human	Hemin	0.1 M NaOH, adjusted to pH 7.4	30 µM	- (whole blood)	*ex vivo*	~200	~150	~25% ↓	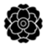	De Souza et al. 2017 [34]
**Coagulation time**	Rabbit	Hematin	0.85% NaCl + 2% Na_2CO_3	25 mg/kg	intravenous	*in vivo*	8 - 11	17	~ x1.8	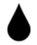	Brown 1913 [3]

**ECLT**	Human	Hematin	alkaline solution 0.1 N NaOH, diluted in PBS	3 nmol	- (plasma sample)	*in vitro*	≥ 10800	2400	~78% ↓	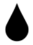	Becker et al. 1985 [24]
**Ethanol gelation**	Human	Heme arginate (Normosang®)	aqueous solution + 40% 1,2-propanediol + 10% ethanol	3 mg/kg	intravenous	*in vivo*	negative	negative[d,e]	No significant change	-	Tenhunen et al. 1987 [16]
Ethanol gelation	Human	Heme arginate (Normosang®)	Physiological saline	3 mg/kg	intravenous	*in vivo*	negative	negative[f]	No significant change	-	Volin et al. 1988 [26]
**FT**	Human (AIP patient)	Hematin (Panhematin®)	0.05 M Na_2CO_3, 0.04 M sorbitol	4 mg/kg	intravenous	Case report	15.5	18.9	~ x1.2	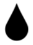	Gajra et al. 2000 [20]
**PT**	Human (AIP patient)	Hematin	N/A	196 mg, every 12 hours	intravenous	Case report	13.2	20.2[a]	~ x1.5	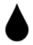	Morris et al. 1981 [11]
PT	Human (AIP patient)	Hematin	N/A	4 mg/kg, every 12 hours	intravenous	Case report	11.7	18.3[b]	~ x1.6	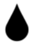	Glueck et al. 1983 [23]
PT	Human (Acute porphyria patient)	Hematin	N/A	4 mg/kg	intravenous	*in vivo*	~4	~10[c]	~ x2.5	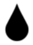	Glueck et al. 1983 [23]
PT	Human	Hematin	1 N NaOH, diluted in potassium phosphat	60 µg/ml	- (plasma sample)	*in vitro*	13.0 (0 h) - 12.0 (50 d)	13.6 (0 h) – 16.4 (50 d)	≤ x1.4 (aged hematin exclusively)	-/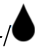	Jones 1986 [25]

			buffer (pH 7.5)								
PT	Human	Hematin	1 N sodium carbonate (pH 7.5))	60 µg/ml	- (plasma sample)	*in vitro*	13.0 (0 h) - 12.0 (50 d)	12.8 (0 h) – 16.1 (50 d)	≤ x1.3 (aged hematin exclusively)	-/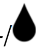	Jones 1986 [25]
PT	Human	Hematin (fresh)	1 M Na_2CO_3, pH 8.0	40 mg/l	- (plasma sample)	*in vitro*	~13.7	~13.3	No significant change	-	Goetsch & Bissell 1986 [35]
PT	Human	Hematin (50 h aged, 21°C)	1 M Na_2CO_3, pH 8.0	40 mg/l	- (plasma sample)	*in vitro*	~13.7	~16.3	~ x1.2	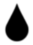	Goetsch & Bissell 1986 [35]
PT	Human	Heme arginate (Normosang®)	Aqueous solution + 40% 1,2-propanediol + 10% ethanol	3 mg/kg	intravenous	*in vivo*	~17.0	~17.3[d], ~17.2[e]	No significant change	-	Tenhunen et al. 1987 [16]
PT	Human	Heme arginate (Normosang®)	Physiological saline	3 mg/kg	intravenous	*in vivo*	~18.0	~18.0 - 19.0[f]	No significant change	-	Volin et al. 1988 [26]
PT	Human	Hematin (Panhematin®)	0.05 M Na_2CO_3, 0.04 M sorbitol	4 mg/kg	intravenous	*in vivo*	N/A	N/A	~ x1.2	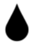	Simionatto et al. 1988 [17]
PT	Human (AIP patient)	Hematin (Panhematin®)	0.05 M Na_2CO_3, 0.04 M sorbitol	4 mg/kg	intravenous	Case report	12	12 (1 hour after 1st injection), 15 (1 hour after 2nd injection, day 4)	If, only very slight change after 2nd injection	-	Green & Ts'ao 1990 [27]

PT	Rat	Albumin-heme (50 g/l & 3 mM, respectively)	0.9% NaCl, pH 7.4	10 - 44% (0.28 - 1.2 mM)	- (whole blood)	*in vitro*	~20.0	~20.0	No significant change	-	Huang et al. 2003 [28]
PT	Rat	Hemin	< 0.5% DMSO	50 mg/kg	intraperitoneal	*in vivo*	~19.3	~20.0	No significant change	-	Desbuards et al. 2007 [29]
PT	Mouse (CLP)	Hemin	DMSO, diluted in PBS	50 µmol/kg	intraperitoneal	*in vivo*	~9.0	~11.0	~ x1.2	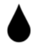	Fei et al. 2012 [30]
PT	Mouse (CLP)	Hemin	DMSO, diluted in PBS	50 µmol/kg	intraperitoneal	*in vivo*	~13.6	~18.6	~ x1.4	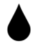	Hassaan et al. 2015 [31]
**RT**	Human (Acute porphyria patient)	Hematin	N/A	4 mg/kg	intravenous	*in vivo*	~20	~30[c]	~ x1.5	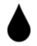	Glueck et al. 1983 [23]
RT	Human (Acute porphyria patient)	Hematin	N/A	0.1 mg/ml	- (plasma sample)	*in vitro*	25	50	~ x2.0	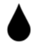	Glueck et al. 1983 [23]
**TT**	Human	Lithium ferriheme	N/A	100 mg. %	- (plasma sample)	*in vitro*	3	N/A	No significant change	-	Barnard 1947 [36]
TT	Human	Lithium ferriheme	N/A	20 mg. %	- (plasma sample)	*in vitro*	3	10	~x3.3	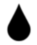	Barnard 1947 [36]
TT	Human	Lithium ferriheme	N/A	40 mg. %	- (plasma sample)	*in vitro*	3	35	~x11.7	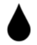	Barnard 1947 [36]
TT	Human	Lithium ferriheme	N/A	60 mg. %	- (plasma sample)	*in vitro*	3	-	Complete clotting prevention	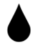	Barnard 1947 [36]

TT	Human (Acute porphyria patient)	Hematin	N/A	4 mg/kg, every 12 hours	intravenous	Case report	N/A	N/A	No significant change[b]	-	Glueck et al. 1983 [23]
TT	Human (Acute porphyria patient)	Hematin	N/A	4 mg/kg	intravenous	*in vivo*	~10	~25[c]	~ x2.5	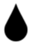	Glueck et al. 1983 [23]
TT	Human (Acute porphyria patient)	Hematin	N/A	0.01-0.03 mg/ml	- (plasma sample)	*in vitro*	~15	~20 - >60	~ x1.3 - >4.0	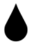	Glueck et al. 1983 [23]
TT	Human	Hematin	0.25% Na_2CO_3; diluted in barbital buffer pH 7.4	0.01 mg/ml	- (plasma sample)	*in vitro*	~13	~46	~ x3.5	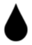	Green et al. 1983 [37]
TT	Human	Hematin	1 N NaOH, diluted in potassium phosphat buffer (pH 7.5)	60 µg/ml	- (plasma sample)	*in vitro*	13.0 (0 h) - 12.0 (50 d)	13.8 (0 h) – 27.8 (50 d)	≤ x2.3 (aged hematin exclusively)	-/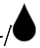	Jones 1986 [25]
TT	Human	Hematin	1 N sodium carbonate (pH 7.5))	60 µg/ml	- (plasma sample)	*in vitro*	13.0 (0 h) - 12.0 (50 d)	12.9 (0 h) – 25.9 (50 d)	≤ x2.2 (aged hematin exclusively)	-/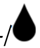	Jones 1986 [25]
TT	Rat	Hematin	1 N sodium carbonate (pH 7.5))	12 mg/kg	intravenous	*in vivo*	14.0	N/A	~ x1.6 (3 min after injection)	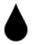	Jones 1986 [25]
TT	Human	Heme arginate (Normosang®)	Aqueous solution + 40% 1,2-	3 mg/kg	intravenous	*in vivo*	~18.7	~18.9[d,e]	No significant change	-	Tenhunen et al. 1987 [16]

			propanediol + 10% ethanol								
TT	Human	Heme arginate (Normosang®)	Physiological saline	3 mg/kg	intravenous	*in vivo*	~19.0	19.0[f]	No significant change	-	Volin et al. 1988 [26]
TT	Human	Hematin (Panhematin®)	0.05 M Na_2CO_3, 0.04 M sorbitol	4 mg/kg	intravenous	*in vivo*	N/A	N/A	~ x1.1	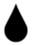	Simionatto et al. 1988 [17]
TT	Human (AIP patient)	Hematin (Panhematin®)	0.05 M Na_2CO_3, 0.04 M sorbitol	4 mg/kg	intravenous	Case report	17	19 (1 hour after 1st injection), 17 (1 hour after 2nd injection, day 4)	No change	-	Green & Ts'ao 1990 [27]
TT	Human (AIP patient)	Hematin (Panhematin®)	0.05 M Na_2CO_3, 0.04 M sorbitol	4 mg/kg	intravenous	Case report	18.9	25.6	~ x1.4	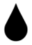	Gajra et al. 2000 [20]

[1] 11 h after first injection. [2] 7 h after 4th injection. [3] 10 min after injection. [d] 15 min after hematin infusion. [e] 4 h after infusion. [f] = 15 min to 24 h after administration. = Evidence for a tendency to an anticoagulant effect of heme and its formulations. ECLT = Euglobulin clot lysis time; PTT = partial thromboplastin time.

Supplementary table S3. Overview of the impact of heme intoxication on cells participating in blood coagulation.

Cell type	Organism	Compound	Solvent	Dose	Injection	Setting	Observation	Effect	Reference
**Platelet**	Rabbit	Hematin	0.85% NaCl + 2% Na_2CO_3 alkaline solution	25 mg/kg	intravenous	*in vivo*	Platelet count ↓ (70% ↓, 1 hour after injection)	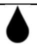	Brown 1913 [3]

Platelet	Human (AIP patient)	Hematin	N/A	196 mg, every 12 hours	intravenous	Case report	Platelet count ↓ (~57% ↓, 12 hours after injection)		Morris et al. 1981 [11]
Platelet	-	Heme	25% pyridine (pH 12.6), diluted with 30% pyridine (pH 12.2)	0.15 - 770 mM	-	*in vitro*	Heme reduction by epinephrine essential for platelet activation		Peterson et al. 1982 [38]
Platelet	-	Hemin	N/A	N/A	-	*in vitro*	Enhancement of ADP- and epinephrine-dependent platelet aggregation, hemin-binding to platelet membrane and granula membrane of platelets		Malik et al. 1983 [39]
Platelet	Human (AIP patient)	Hematin	N/A	4 mg/kg, every 12 hours	intravenous	Case report	Platelet count ↓ (~12% ↓, 7 hours after 4th injection), thrombocytopenia	-	Glueck et al. 1983 [23]
Platelet	Human (AIP patient)	Hematin	N/A	4 mg/kg	intravenous	*in vivo*	Platelet count ↓ (~41% ↓, 10 minutes after injection)	-	Glueck et al. 1983 [23]
Platelet	Human	Hematin	N/A	0.1 mg/ml (≙ 158 µM)	- (platelet-rich plasma sample)	*in vitro*	Platelet aggregation ↑, Serotonin secretion ↑, ATP secretion ↑		Glueck et al. 1983 [23]
Platelet	Human	Hematin	0.25% Na_2CO_3 (pH 7.4), diluted with isotonic saline	2 - 5 µg/ml (≙ 3.2 - 7.9 µM)	- (washed platelets)	*in vitro*	Platelet aggregation ↑		Neely et al. 1984 [40]
Platelet	Human	Hematin	0.25% Na_2CO_3 (pH 7.4), diluted with isotonic saline	5 - 10 µg/ml (≙ 7.9 - 15.8 µM)	- (washed platelets)	*in vitro*	Thromboxane A_2 generation ↑ (~ 92.7 - 187.3 ng per billion platelets; dose-dependent)		Neely et al. 1984 [40]

Platelet	Human	Hematin	0.25% Na_2CO_3 (pH 7.4), diluted with isotonic saline	2 µg/ml (≙ 3.2 µM)	- (washed platelets)	*in vitro*	ATP release ↓ (slightly)	❁	Neely et al. 1984 [40]
Platelet	Human (myelodysplastic syndrome patients)	Heme arginate (Normosang®)	0.9% saline solution	2 - 3 mg/kg (applied as 25 mg/ml, repetitive)	intravenous	Case reports	Platelet count ↑ (~ 2.2 - >8-fold after several weeks)	-	Ruutu et al. 1987 [15], Volin et al. 1988 [41]
Platelet	Human (AIP patients)	Heme arginate (Normosang®)	0.9% NaCl	3 mg/kg	intravenous	Case reports	Platelet count ↑ (~ 1.2-fold after several weeks)	-	Herrick et al. 1989 [18]
Platelet	Human (AIP patient)	Hematin (Panhematin®)	0.05 M Na_2CO_3, 0.04 M sorbitol	4 mg/kg	intravenous	Case report	Platelet count ↑ (e.g., ~ 1.2-fold, 24 hours after treatment), platelet aggregation ↓, Platelet ATP content ↓, platelet ADP content ↓	-	Green & Ts'ao 1990 [27]
Platelet	Human (AIP patient)	Hematin (Panhematin®)	0.05 M Na_2CO_3, 0.04 M sorbitol	4 mg/kg	intravenous	Case report	Platelet count ↓ (44% ↓)		Gajra et al. 2000 [20]
Platelet	Rat	Albumin-heme (50 g/l & 3 mM, respectively)	0.9% NaCl, pH 7.4	10 - 44% (≙ 0.28 - 1.2 mM)	- (whole blood)	*in vitro*	No change of total number	-	Huang et al. 2003 [28]
Platelet	Mouse (wild-type & HO-1 KO)	Hemin	Phosphate-buffered saline	15 - 40 mg/kg, twice daily	intraperitoneal	*in vivo*	Platelet cGMP level ↑, accelerated platelet-rich thrombi formation in HO-1 knockout mice after induction	❁/-	Peng et al. 2004 [42]

							by $FeCl_3$, no thrombosis promotion by heme without $FeCl_3$ pretreatment		
Platelet	Rat	Hemin	< 0.5% DMSO	50 mg/kg	intraperitoneal	*in vivo*	Significant increase of platelet count (~1.4-fold)	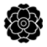	Desbuards et al. 2007 [29]
Platelet	Human	Albumin-heme (50 g/l & 3 mM, respectively)	0.9% NaCl, pH 7.4	10 - 40% (≙ 0.28 - 1.1 mM)	- (whole blood)	*in vitro*	No change of ADP-stimulated platelet activation	-	Komatsu et al. 2007 [43]
Platelet	Mouse	Hemin	N/A	1 mM	- (mouse aorta)	*ex vivo*	Platelet aggregation as a consequence of endothelial denudation ↑	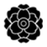	Woollard et al. 2009 [44]
Platelet	Human	Hemin	N/A	0 - 25 μM	- (platelets)	*in vitro*	Ferroptosis ↑, platelet activation ↑, P-selectin translocation ↑, reorganization of actin filaments ↑, lipid peroxidation ↑, cell viability ↑, LDH release ↑, HO-1 expression ↑, cytosolic iron ↑, GSH/GSSG ratio ↓, γ-glutamyltransferase level ↑	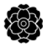	NaveenKumar et al. 2018 [45]
Platelet	Human	Hemin	N/A	0-25 μM	- (platelets)	*in vitro*	Activation of platelets ↑, Cell viability ↓, LDH release ↑, P-selectin level ↑, filopodia-like structure of platelets (10 μM hemin), ROS generation ↑, mitochondrial cardiolipin oxidation ↑, mitochondrial membrane potential ↓, anti-apoptotic Bcl-2 level ↓, apoptotic Bcl-2 level ↑, GSH/GSSG ratio ↓, NLRP-3 activation ↑, prevention by melatonin	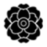	NaveenKumar et al. 2019 [46]

Platelet	Mouse	Hemin	N/A	50 µmol/kg	N/A	*in vivo*	Platelet number ↓, soluble P-selectin level ↑, prevention by melatonin		NaveenKumar et al. 2019 [46]
Platelet	Human	Hemin	N/A	0.75 - 50 µM	- (platelets)	*in vitro*	Rapid platelet aggregation (2 - 12 µM), slower/reduced platelet aggregation (25 µM), P-selectin expression ↑, GPIIbIIIa activation ↑, phosphatidylserine exposure on platelets (≥ 25 µM), phosphorylation of Syk and PLCγ2 ↑, CLEC2-mediated platelet activation, independent from oxidative stress		Bourne et al. 2020 [47]
Platelet	Mouse	Hemin	N/A	1.56 - 50 µM	- (platelets)	*in vitro*	Platelet aggregation ↑, CLEC2-mediated platelet activation		Bourne et al. 2020 [47]
**Endothelial cell**	Bovine	Hematin	0.25% Na_2CO_3 (pH 7.4), diluted with isotonic saline or 50 mM HEPES buffer	2 - 40 µg/ml (≙ 3.2 - 63.1 µM)	- (BAECs)	*in vitro*	Reversible morphologic changes of BAECs (bulging, exposed areas, surface vesiculation, cell retraction), no significant change in cell detachment		Neely et al. 1984 [48]
Endothelial cell	Bovine	Hematin	0.25% Na_2CO_3 (pH 7.4), diluted with isotonic saline or 50 mM HEPES buffer	100 µg/ml (≙ 158 µM)	- (BAECs)	*in vitro*	Cell detachment ↑ (from ~7.9 to ~13.0%)		Neely et al. 1984 [48]

Endothelial cell	Bovine	Hematin	0.25% Na_2CO_3 (pH 7.4), diluted with isotonic saline or 50 mM HEPES buffer	40 μg/ml (≙ 63.1 μM)	- (BAECs)	*in vitro*	Platelet adhesion to BAECs ↑ (2-fold)	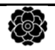	Neely et al. 1984 [48]
Endothelial cell	Pig	Hemin	20 mM NaOH, diluted with buffer	N/A	- (PAECs)	*in vivo*	Rapid uptake of heme, sensitivity towards oxidants ↑	-	Balla et al. 1991 [49]
Endothelial cell	Pig	Hemin	20 mM NaOH, diluted with buffer	1 - 10 μM	- (PAECs)	*in vivo*	LDL oxidation ↑; extremely cytotoxic	-	Balla et al. 1991 [50]
Endothelial cell	Mouse	Hemin	N/A	1 mM	- (mouse aorta)	*ex vivo*	Denudation of endothelium ↑, collagen exposure ↑, platelet aggregation ↑	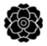	Woollard et al. 2009 [44]
Endothelial cell	Bovine	Hemin	DMSO	10 - 50 μM	- (BAECs)	*in vitro*	LDH release ↑, apoptosis ↑, ATP ↓ (only when 50 μM hemin was applied), mitochondrial dysfunction ↑, lipid peroxidation ↑, protein carbonyl formation ↑, protein thiol oxidation ↑, autophagy activation ↑, mitophagy induction ↑		Higdon et al. 2012 [51]
Endothelial cell	Mouse (NY1DD)	Hemin (as Panhematin®)	D-sorbitol, Na_2CO_3, saline (as Panhematin®)	0.4 - 32 μmol/kg	intravenous	*in vivo*	Endothelial cell activation through TLR4 signaling ↑, vaso-occlusion ↑, expression of adhesion molecules ↑	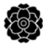	Belcher et al. 2014 [52]

Endothelial cell	Human	Hemin (as Panhematin®)	D-sorbitol, Na_2CO_3, saline (as Panhematin®)	20 μM	- (HUVECs)	*in vitro*	Endothelial cell activation through TLR4 signaling ↑, vaso-occlusion ↑, expression of adhesion molecules ↑		Belcher et al. 2014 [52]
Endothelial cell	Human	Heme-laden MPs (SCD)	N/A	~20 nM (50 control MPs/μl), ~65 nM (50 SCD MPs/μl)	- (HUVECs)	*in vitro*	Transfer of heme to HUVECs by MPs (SCD MPs & synthetic heme-laden MLVs: transfer ↑ (~4-fold), Blockage of transfer via annexin-a5 (PS-antagonist, 10 μg/ml) or hemopexin (2 μM), ROS production ↑ (inhibited by annexin-a5, reduced by hemopexin (50%), prevented by TLR4 neutralization), apoptosis ↑		Camus et al. 2015 [53]
Endothelial cell	Mouse (SAD)	Heme-laden MPs (SCD)	N/A	300 MPs/μl	intravenous	*ex vivo*	ACH-dependent vasodilation of endothelium ↓, blood flow velocity in renal arteries ↓ (30% ↓)		Camus et al. 2015 [53]
Endothelial cell	Mouse (SAD)	Heme	N/A	100 nM	intravenous	*ex vivo*	ACH-dependent vasodilation of endothelium ↓		Camus et al. 2015 [53]
Endothelial cell	Human	Hemin	0,25 M NaOH, diluted in PBS (pH 7.4)	5 - 100 μM	- (HLMVECs)	*in vitro*	Transendothelial electrical resistance ↓, ermeability of monolayer ↑, programmed cell death ↑, effects abrogable by: TLR4-inhibitor, antioxidant N-acetylcystein, iron chelator deferoxamine), MLKL activation (necroptosis) ↑	-	Singla et al. 2017 [54]
Endothelial cell	Human	Hemin	50 mM NaOH, 145 mM NaCl, diluted with medium	12.5 - 50 μM	- (HUVECs)	*in vitro*	C3 deposition ↑, TM expression ↑, HO-1 gene expression ↑, HO-1 protein expression ↑		May et al. 2018 [55]

Endothelial cell	Human	Hemin	50 mM NaOH, 145 mM NaCl, diluted with medium	12.5 - 50 µM	- (HMECs)	*in vitro*	C3 deposition ↑, TM expression ↑, HO-1 gene expression ↑, HO-1 protein expression ↑		May et al. 2018 [55]
Endothelial cell	Human	Hemin	50 mM NaOH, 145 mM NaCl, diluted with medium	12.5 - 50 µM	- (GENCs)	*in vitro*	C3 deposition ↑, TM expression ↑, HO-1 gene expression ↑, HO-1 protein expression ↑ (slightly)		May et al. 2018 [55]
Endothelial cell	Human	Hemin	50 mM NaOH, 145 mM NaCl, diluted with medium	12.5 - 50 µM	- (HRGECs)	*in vitro*	C3 deposition ↑ (higher extent than in vascular ECs), TM expression ↑, HO-1 gene expression ↑, HO-1 protein expression ↑ (slightly)		May et al. 2018 [55]
Endothelial cell	Mouse (C57B1/6)	Hemin	50 mM NaOH, 145 mM NaCl, diluted with PBS	40 µmol/kg	intraperitoneal	*in vivo*	C3 deposition in kidney glomeruli ↑, skin and large liver vessel TM levels ↑, lung microvasculature TM levels ↓, HO-1 levels in proximal tubules (liver, heart, skin, lungs) ↑, HO-1 colocalization with blood vessels		May et al. 2018 [55]
Endothelial cell	Human	Hemin	50 mM NaOH, 145 mM NaCl, diluted with vehicle	50 µM	- (HUVECs)	*in vitro*	P-selectin expression ↑, complement activation ↑, protection by hemopexin		Merle et al. 2018 [56]
Endothelial cell	Human	Hemin	0.1 M NaOH, diluted in FBS	20 - 40 µM	- (ECs)	*in vitro*	P-selectin expression ↑, VCAM-1 expression ↑, HbS RBC adhesion to heme-activated ECs ↑		Kucukal et al. 2018 [57]
**RBC**	Rabbit	Hematin	0.85% NaCl + 2% Na_2CO_3	< 15 mg/kg	Intravenous	*in vivo*	Rarely: RBC count ↓ (fast regeneration)	-	Brown 1913 [3]

			alkaline solution						
RBC	Rabbit	Hematin	0.85% NaCl + 2% Na_2CO_3 alkaline solution	20 mg/kg	Intravenous	*in vivo*	RBC count ↓ (decrease of ≥ 1000000 cells)	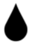	Brown 1913 [3]
RBC	Rabbit	Hematin	0.85% NaCl + 2% Na_2CO_3 alkaline solution	10 mg/kg (daily)	intravenous	*in vivo*	Irregular size, variable color, presence of immature cells (in particular basophilic cells), RBC count ↓ (~60%)	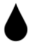	Brown 1913 [3]
RBC	Human (AIP patient)	Hematin	N/A	196 mg, every 12 hours	intravenous	Case report	Hematocrit ↓ (from 32% to 22%, 12 hours after injection)	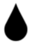	Morris et al. 1981 [11]
RBC	Mouse	Heme	5 mM NaOH, diluted with medium	0 - 7 µM	- (RBCs)	*in vitro*	Hemolysis ↑, glutathione level ↓, ATP level ↓, hemoglobin amount ↓	-	Chou et al. 1981 [58]
RBC	Human (AIP patient)	Hematin	N/A	4 mg/kg, every 12 hours	intravenous	Case report	Hematocrit ↓ (from 36% to 32%, 7 hours after 4th injection), no morphological changes	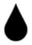	Glueck et al. 1983 [23]
RBC	Human	Hemin	0.01 M NaOH, diluted with water	0 - 500 µM	- (RBCs)	*in vitro*	Conformational change of spectrin and protein 4.1, RBC membrane stability ↓	-	Liu et al. 1985 [59]
RBC	Human	Hemin	20 mM NaOH, diluted with water	0.2 - 40 µM	- (RBCs)	*in vitro*	Hemolysis ↑, heme accumulation in RBC membrane, heme extractable from membranes by albumin		Shaklai et al. 1985 [60]
RBC	Rat	Albumin-heme (50 g/l & 3 mM,	0.9% NaCl, pH 7.4	10 - 44% (0.28 - 1.2 mM)	- (whole blood)	*in vitro*	No change	-	Huang et al. 2003 [28]

		respectively)							
RBC	Rat	Hemin	< 0.5% DMSO	50 mg/kg	intraperitoneal	*in vivo*	No significant change of hematocrit, no change of RBC count	-	Desbuards et al. 2007 [29]
**Leukocyte**	Guinea pig	Hematin, hemin	0.85% salt solution	30 - 54 mg/kg	intraperitoneal, subcutaneous, intravenous	*in vivo*	Polymorphonuclear leucocyte ↑	-	Brown 1911 [1]
Leukocyte	Rabbit	Hematin	0.85% NaCl + 2% Na_2CO_3 alkaline solution	10 mg/kg	intravenous	*in vivo*	Leukocytosis; total leukocyte number ↑ (~1.4-fold), large (~5.6-fold) and small (~1.5-fold) mononuclear cells ↑, basophiles ↑ (~2.7-fold), eosinophils ↓ (decrease of ~60%)	-	Brown 1913 [3]
Leukocyte	Rabbit	Hematin	0.85% NaCl + 2% Na_2CO_3 alkaline solution	20 mg/kg (daily)	intravenous	*in vivo*	marked leukocytosis; total leukocyte number ↑ (x ~4.6), large mononuclear cells ↑ (~10-fold), polymorphonuclear cells ↑ (~7.8-fold)	-	Brown 1913 [3]
Leukocyte	Human (myelodysplastic syndrome patients)	Heme arginate (Normosang®)	0.9% saline solution	2 - 3 mg/kg (applied as 25 mg/ml, repetitive)	intravenous	Case reports	Neutrophil count ↑ (~2.1 - >5-fold)	-	Ruutu et al. 1987 [15], Volin et al. 1988 [41]
Leukocyte	Human	Hematin	N/A	N/A	- (PBMC)	*in vitro*	Procoagulant activity of PBMCs ↑		Smith & Winslow 1992 [61]
Leukocyte	Mouse	Hemin	0.1 N NaOH, 10 mM Tris base (pH 8.6)	750 µM (intravascular conc.)	intravenous	*in vivo*	Leukocyte accumulation ↑ (spleen, liver, kidney, intestines, femur, brain, pancreas)	-	Wagener et al. 2001 [62]

Leukocyte	Human	Hemin	DMSO, diluted with PBS	0.1 - 20 μM	- (PMNs)	*in vitro*	Neutrophil recruitment ↑ (PKC activation required), actin polymerization ↑, oxidative burst in neutrophils ↑, PKC activation ↑, IL-8 expression ↑	-	Graça-Souza et al. 2002 [63]
Leukocyte	Rat	Hemin	DMSO, diluted with PBS	30 – 300 nmol/cavity	intrathoracic	*in vivo*	Neutrophil migration ↑, pleural neutrophil population ↑, no changes of mononuclear cells	-	Graça-Souza et al. 2002 [63]
Leukocyte	Rat	Albumin-heme (50 g/l & 3 mM, respectively)	0.9% NaCl, pH 7.4	10 - 44% (≙ 0.28 - 1.2 mM)	- (whole blood)	*in vitro*	No change	-	Huang et al. 2003 [28]
Leukocyte	Rat	Hemin	with Trizma base in 0.1 M NaOH, diluted in saline, pH 8	750 μM	intradermally	*in vivo*	Leukocyte recruitment ↑	-	Wagener et al. 2003 [64]
Leukocyte	Human	Hemin	DMSO, diluted with PBS	1 - 50 μM	- (neutrophils)	*in vitro*	Delay in neutrophil apoptosis, de novo synthesis of antiapoptotic proteins (e.g., Bcl-xL), ERK-2 translocation ↑, Akt phosphorylation ↑, degradation of proapoptotic proteins (e.g. Bad), NF-κB activation ↑	-	Arruda et al. 2004 [65]
Leukocyte	Rat	Hemin	< 0.5% DMSO	50 mg/kg	intraperitoneal	*in vivo*	Higher total leucocyte count (x ~1.9), no changes of lymphocyte cell number	-	Desbuards et al. 2007 [29]

Leukocyte	Mouse (NY1DD)	Hemin (as Panhematin®)	D-sorbitol, Na_2CO_3, saline (as Panhematin®)	3.2 µmol/kg	intravenous	*in vivo*	Leukocyte rolling ↑, leukocyte adhesion ↑ (TLR4-dependent)	-	Belcher et al. 2014 [52]
Leukocyte	Mouse (SA)	Hemin	DMSO, diluted in Hanks balanced salt solution	50 µmol/kg	intraperitoneal	*in vivo*	NET formation ↑	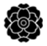	Chen et al. 2014 [66]
Leukocyte	Human	Hemin	DMSO, diluted in Hank's balanced salt solution	10 - 20 µM	- (BM neutrophils)	*in vitro*	NET formation ↑	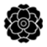	Chen et al. 2014 [66]
Leukocyte	Human	Hemin	N/A	1.5 - 38.3 µM	- (neutrophils)	*in vitro*	NET formation ↑	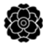	Ohbuchi et al. 2017 [67]

= Evidence for a tendency to an anticoagulant effect of heme and its formulations. 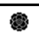 = Evidence for a tendency to a procoagulant effect of heme and its formulations. ACH = acetylcholine; BAECs = Bovine aortic endothelial cells; BM = Bone marrow; FBS = fetal bovine serum; GENCs = Glomerular endothelial cells; HLMVECs = human lung microvascular endothelial cells; HMECs = Human Mammary Epithelial Cells; HO-1 = heme oxygenase 1; HRGECs = Human renal glomerular endothelial cells; HUVECs = human umbilical vein endothelial cells; MLKL = mixed lineage kinase domain-like; MLVs = synthetic multilayer vesicles; MPs = erythrocyte membrane microparticles; N/A = not applicable; n.d. = not determined; PMNs = Polymorphonuclear neutrophils, SA = hemizygous mice: Tg[Hu-miniLCRα1GγAγδβS] Hba−/−Hbb+/−; SAD = transgenic S-Antilles-D Punjab Hb-expressing mouse model of SCD; TM = thrombomodulin.

Supplementary table S4. Overview of the impact of heme and its formulations on proteins acting in blood coagulation.

Protein	Organism	Compound	Solvent	Dose	Injection	Setting	Observation	Effect	Reference
**Activated protein C**	Mouse (CLP)	Hemin	DMSO, diluted in PBS	50 µmol/kg	intraperitoneal	*in vivo*	APC plasma level ↑ (4 - 24 h after treatment)	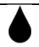	Fei et al. 2012 [30]
Activated protein C	Human	Hemin	30 mM NaOH, diluted in Tris-	187.5 - 3000 nM	-	*in vitro*	K_D of 400 ± 47 nM, stoichiometry: 1:1.3	-	Hopp et al. 2020 [32]

			HCl buffer (pH 7.4)				(APC:heme), two binding sites identified		
Activated protein C	Human	Hemin	30 mM NaOH, diluted in HEPES buffer (pH 7.0)	0 - 50 μM	-	*in vitro*	Amidolytic activity of rAPC (50 nM) and pdAPC (100 nM) ↓, K_i (pdAPC) = 12.56 ± 2.31 μM	-	Hopp et al. 2020 [32]
Activated protein C	Human	Hemin	30 mM NaOH, diluted in DPBS buffer (pH 7.4)	0 - 100 μM	-	*in vitro*	Anticoagulant activity of APC (5 nM) ↓	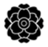	Hopp et al. 2020 [32]
Activated protein C	Human	Hemin	30 mM NaOH, diluted in medium	120 μM	- (HUVECs)	*in vitro*	APC (20 nM) protects from heme's cytotoxicity	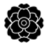	Hopp et al. 2020 [32]
**α2-antiplasmin**	Human	Heme	-	-	-	-	Suggestion of a permanently bound heme	-	Arkebauer et al. 2011 [68]
**Antithrombin-III**	Human (AIP patient)	Hematin	N/A	4 mg/kg	intravenous	*in vivo*	No change of plasma level	-	Glueck et al. 1983 [23]
Antithrombin-III	Human	Heme arginate (Normosang ®)	physiological saline	3 mg/kg	intravenous	*in vivo*	Plasma levels not altered	-	Volin et al. 1988 [26]
**Collagen**	Mouse	Hemin	N/A	1 mM	- (mouse aorta)	*ex vivo*	Endothelial collagen expression ↑	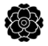	Woollard et al. 2009 [44]
**E-selectin**	Human	Hemin	N/A	100 μM	- (HUVECs)	*in vitro*	Surface expression of E-selectin (4-fold) ↑	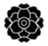	Wagener et al. 1997 [69]
**Factor V**	Human (AIP patient)	Hematin	N/A	4 mg/kg	intravenous	*in vivo*	Plasma level ↓/FV activity ↓ (~80%)[a]	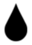	Glueck et al. 1983 [23]
**Factor VIII**	Human (AIP patient)	Hematin	N/A	4 mg/kg	intravenous	*in vivo*	Plasma level ↓ (~36.8%) [a]	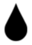	Glueck et al. 1983 [23]

Factor VIII	Human (AIP patient)	Hematin	N/A	4 mg/kg	intravenous	*in vivo*	Plasma level ↓ (~36.8%) [a]	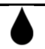	Glueck et al. 1983 [23]
Factor VIII	Human	Hematin	0.25% Na_2CO_3 (24h), diluted with barbital buffer (pH 7.4)	18 - 70 µg/ml	- (plasma)	*in vitro*	FVIII procoagulant activity ↓ (72% in pooled plasma, 88% in FVIII concentrate)	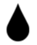	Green et al. 1983 [37]
Factor VIII	Human	^{59}Fe- and ^{3}H-labeled Hematin	0.25% Na_2CO_3	e.g. 0.17 mg/ml	-	*in vitro*	Formation of FVIII/VWF complex, inhibition of FVIII-VWF dissociation, binding of FVIII/VWF-hematin complex to platelets	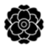	Green et al. 1986 [70]
Factor VIII	Human	Hemin	DMSO, diluted in buffer	0.01 - 6.4 µM	-	*in vitro*	Specific heme binding to rFVIII (Soret band shift to ~412 nm), ~10 binding sites, K_D(full-length rFVIII) = 12.7 ± 6.1 nM, K_D(B-domain-deleted rFVIII) = 1.9 ± 0.5 nM	-	Repessé et al. 2012 [71]
Factor VIII	Human	Hemin	DMSO, diluted in buffer	Molar excess	-	*in vitro*	FVIII procoagulant activity ↓ (full-length rFVIII: by up to ~50%; B-domain-deleted rFVIII: by up to ~51%), prevention by VWF	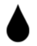	Repessé et al. 2012 [71]
Factor VIII	Human	Hemin	DMSO, diluted in buffer	Molar excess	-	*in vitro*	FVIII-VWF interaction not altered, FVIII-phosphatidyl serine interaction not altered, FVIII-activated platelet interaction not altered, thrombin can cleave heme-bound FVIII	-	Repessé et al. 2012 [71]

Factor VIII	Human	Hemin	DMSO, diluted in buffer	Molar excess	-	*in vitro*	Impaired FVIII-FIX interaction (by ~52%)	💧	Repessé et al. 2012 [71]
**Factor XII**	Human	Hematin	0.1 N NaOH, diluted in PBS	3 - 24 nmol	- (plasma)	*in vitro*	Increased activation (amidolytic and autoactivation) suggested	❁	Becker et al. 1985 [24]
Factor XII	Mouse	Hemin	0.1 M NaOH and 0.1 M Tris-HCl (pH 8.0)	0 - 35 µmol/kg	Retro-orbital	*in vivo*	Blocking of intrinsic pathway via FXII did not influence heme-driven coagulation activation	-	Sparkenbaugh et al. 2015 [72]
**Factor XIIIa**	Human	Hemin	30 mM NaOH, diluted in Tris buffer (pH 7.5)	0 - 50 µM	-	*in vitro*	No impact on the amidolytic activity of rFXIIIa (400 nM)	-	Hopp et al. 2020 [32]
**FDP**	Human (AIP patient)	Hematin	N/A	4 mg/kg	intravenous	*in vivo*	Plasma level ↑ (~ x2) [a]	💧	Glueck et al. 1983 [23]
FDP	Human	Heme arginate (Normosang ®)	physiological saline	3 mg/kg	intravenous	*in vivo*	Plasma levels not altered	-	Volin et al. 1988 [26]
**Fibrinogen**	Human (AIP patient)	Hematin	N/A	4 mg/kg	intravenous	*in vivo*	Plasma level ↓ (~32.1 %) [a]	💧	Glueck et al. 1983 [23]
Fibrinogen	Bovine	Hematin	0.25% Na_2CO_3 (24h), diluted with barbital buffer (pH 7.4)	50 µg/ml	-	*in vitro*	Hematin binding to fibrinogen (2 mg/ml), thrombin clottablity of fibrinogen ↓	💧	Green et al. 1983 [37]
Fibrinogen	Human	Hematin	0.25% Na_2CO_3 (pH 7.4), diluted with isotonic saline	7 µg/ml	- (gel-filtered platelets)	*in vitro*	Hematin-induced fibrinogen binding to platelets ↑ (~13.8%; 30 min preincubation)	❁	Neely et al. 1984 [40]
Fibrinogen	Human	Heme arginate	physiological saline	3 mg/kg	intravenous	*in vivo*	Plasma levels not altered	-	Volin et al. 1988 [26]

		(Normosan g ®)							
Fibrinogen	Human	Heme	-	-	-	*in vitro*	Suggestion of at least one permanently bound heme	-	Nielsen et al. 2011 [73]
Fibrinogen	Human	Hemin	0.1 M NaOH, diluted with T buffer	10 μM	-	*in vitro*	Binding of hemin to immobilized fibrinogen	-	Orino 2013 [74]
Fibrinogen	Pig	Hematin	0.05 M NaOH	0 - 500 μM	- (blood, plasma)	*in vitro*	Cross-linking (≥ 1 min exposure) ↑, formation of high molecular weight polymers (> 245 kDa) ↑, H_2O_2-dependent	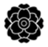	Ke & Huang 2016 [75]
Fibrinogen	Human	Hematin	0.05 M NaOH	30 μM	-	*in vitro*	Cross-linking of fibrinogen (10 mg/ml; 2 min exposure) ↑, membrane-like layer formation ↑, formation of high molecular weight polymers (> 245 kDa) ↑, dityrosine formation ↑	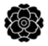	Ke & Huang 2016 [75]
Fibrinogen	Human	Hematin	0.05 M NaOH	20 μM	-	*in vitro*	Binding to fibrinogen (Soret band shift to 410 nm; hexacoordination)	-	Ke & Huang 2016 [75]
Fibrinogen	Human	Hemin	DMSO	25 - 50 μM	-	*in vitro*	Binding to fibrinogen (Soret band red-shift, change of conformation), cross-linking of fibrinogen, one binding site predicted, K_b = 3.0 ± 0.4 x 10^5 M^{-1}, increased peroxidase-like activity of hemin	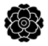	Hou et al. 2018 [76]
**Fibrin**	Human	Hematoporphyrin	N/A	N/A	-	*in vitro*	No binding to fibrin	-	Musser et al. 1980 [77]

**ICAM-1**	Human	Hemin	N/A	50 - 100 μM	- (HUVECs)	*in vitro*	Surface expression of ICAM-1 (2 to 4.4-fold) ↑		Wagener et al. 1997 [69]
ICAM-1	Mouse	Hemin	0.1 N NaOH, 10 mM Tris base (pH 8.6)	750 μM	intravenous	*in vivo*	Endothelial surface expression of ICAM-1 ↑ (1 - 24 hours after administration)		Wagener et al. 2001 [62]
**Kallikrein**	Human	Hematin	0.1 N NaOH, diluted in PBS	3 - 24 nmol	- (plasma)	*in vitro*	Amidolytic activity ↑ (e.g., ~10-fold faster conversion of substrate in presence of 12 nmol hematin)		Becker et al. 1985 [24]
**Plasmin**	Pig	Hematin	0.25% Na_2CO_3 (24h), diluted with barbital buffer (pH 7.4)	0.006 - 0.09 μg/ml	-	*in vitro*	Amidolytic activity ↓ (6 - 50%; 1 min preincubation)		Green et al. 1983 [37]
Plasmin	Pig	Hematin	0.25% Na_2CO_3 (24h), diluted with barbital buffer (pH 7.4)	15 μg/ml	- (whole blood clot)	*in vitro*	Inhibition of clot lysis		Green et al. 1983 [37]
Plasmin	Human	Heme	-	-	-	*in vitro*	Suggestion of a permanently bound heme	.	Arkebauer et al. 2011 [68]
**Plasminogen**	Human (AIP patient)	Hematin	N/A	4 mg/kg	intravenous	*in vivo*	No change of plasma level	-	Glueck et al. 1983 [23]
**Protein C**	Mouse (CLP)	Hemin	DMSO, diluted in PBS	50 μmol/kg	intraperitoneal	*in vivo*	Protein C plasma level ↑ (4 - 24 h after treatment)		Fei et al. 2012 [30]
**P-selectin**	Human	Hemin	N/A	10 μM	- (HUVECs)	*in vitro*	TLR-4-dependent surface P-selectin expression ↑		Belcher et al. 2011 [78]
P-selectin	Human	Hemin	50 mM NaOH / 145 mM NaCl, diluted in medium	100 μM	- (HUVECs)	*in vitro*	Membrane P-selectin expression ↑		Frimat et al. 2013 [79]
P-selectin	Human	Hemin	D-sorbitol, Na_2CO_3, saline	20 μM	- (HUVECs)	*in vitro*	P-selectin expression ↑		Belcher et al. 2014 [52]

		(as Panhematin®)	(as Panhematin®)						
P-selectin	Mouse (C57BL/6, NY1DD)	Hemin (as Panhematin®)	D-sorbitol, Na_2CO_3, saline (as Panhematin®)	3.2 µmol/kg	intravenous	*in vivo*	P-selectin expression on vessel walls ↑, dependent on NOX, PKC and oxidants, TLR-4-mediated		Belcher et al. 2014 [52]
P-selectin	Mouse	Hemin	0.1 N NaOH, 10 mM Tris base (pH 8.6)	750 µM	intravenous	*in vivo*	Endothelial surface expression of P-selectin ↑ (one hour after administration)		Wagener et al. 2001 [62]
**Thrombin**	Human	Hematin	N/A	70 µg/ml	- (plasma)	*in vitro*	Time- and temperature-dependent prolongation of TT		Glueck et al. 1983 [23]
Thrombin	Human	Hematin (4 weeks aged)	carbonate buffer (pH 7.4)	6 µg/ml	-	*in vitro*	Inhibition of thrombin (0.25 U/ml)-mediated fibrinopeptide A generation		Jones et al. 1986 [80]
Thrombin	Bovine	Hematin	0.25% Na_2CO_3 (24h), diluted with barbital buffer (pH 7.4)	50 µg/ml	-	*in vitro*	Hematin binding to thrombin (2 mg/ml),	-	Green et al. 1983 [37]
Thrombin	Human	Hematin	0.25% Na_2CO_3 (24h), diluted with barbital buffer (pH 7.4)	0 - 0.238 µg/ml	-	*in vitro*	amidolytic activity of α-thrombin (17.4 - 69.6 µU/ml) ↓ (by 13 – 86.3%)		Green et al. 1983 [37]
Thrombin	Mouse	Hemin	0.1 M NaOH and 0.1 M Tris-HCl (pH 8.0)	0 - 35 µmol/kg	Retro-orbital	*in vivo*	Thrombin-antithrombin complex plasma levels ↑ (up to 2.7-fold)		Sparkenbaugh et al. 2015 [72]
Thrombin	Human	Hemin	30 mM NaOH, diluted in HEPES buffer (pH 7.0)	0 - 50 µM	-	*in vitro*	No impact on the amidolytic activity of α-thrombin (25 nM)	-	Hopp et al. 2020 [32]

**Thrombomodulin**	Mouse (CLP)	Hemin	DMSO, diluted in PBS	50 µmol/kg	intraperitoneal	*in vivo*	Thrombodulin plasma level ↓ (4 - 24 h after treatment)		Fei et al. 2012 [30]
**Tissue factor**	Human	Hemin	0.05 M NaOH, diluted in medium	1 - 100 µM	- (ECs)	*in vitro*	Dose-dependent upregulation of TF expression ↑ (up to 50-fold with 100 µM heme)		Setty et al. 2008 [81]
Tissue factor	Human	Hemin	0.05 M NaOH, diluted in medium	100 µM	- (ECs)	*in vitro*	Time-dependent upregulation of TF mRNA expression ↑ (17-fold), time-dependent upregulation of total endothelial TF protein level (after 4 hours; functionally active TF) ↑		Setty et al. 2008 [81]
Tissue factor	Human	Hemin	N/A	10 µM	- (plasma)	*in vitro*	TF level in PBMCs and monocytes ↑, prevention by hemopexin (15 µM), TF mRNA expression in monocytes ↑		Rehani et al. 2013 [82]
Tissue factor	Human	Hemin	N/A	30 µM	- (plasma)	*in vitro*	TF expression in PBMCs ↑		Souza et al. 2014 [83]
Tissue factor	Human/ Mouse	Hemin	0.1 M NaOH and 0.1 M Tris-HCl (pH 8.0)	5- 50 µM	-	*in vitro*	TF expression in PBMCs and RAW 264.7 macrophages ↑		Sparkenbaugh et al. 2015 [72]
Tissue factor	Mouse	Hemin	0.1 M NaOH and 0.1 M Tris-HCl (pH 8.0)	35 µmol/kg	Retro-orbital	*in vivo*	Blocking of intrinsic pathway did not influence heme-driven procoagulant activity → important role for extrinsic, TF-mediated pathway		Sparkenbaugh et al. 2015 [72]
Tissue factor	Human	Hemin	0.1 M NaOH, adjusted to pH 7.4	30 µM	- (whole blood)	*ex vivo*	TF antibody inhibited heme-induced coagulation		De Souza et al. 2017 [34]

							→ TF-dependency of heme's procoagulant effects		
Tissue factor	Human	Hemin	0.1 M NaOH, diluted with water	5 - 30 µM	- (PBMCs)	*in vitro*	TF expression (≥ 10 µM hemin) ↑	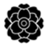	Hounkpe et al. 2020 [84]
Tissue factor	Mice	Hemin	50 mM NaOH, 145 mM NaCl	100 µmol/kg	intraperitoneal	*in vivo*	TF expression ↑	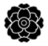	May et al. 2020 [85]
**VCAM-1**	Human	Hemin	N/A	100 µM	- (HUVECs)	*in vitro*	Surface expression of VCAM-1 (3-fold) ↑	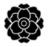	Wagener et al. 1997 [69]
VCAM-1	Mouse	Hemin	0.1 N NaOH, 10 mM Tris base (pH 8.6)	750 µM	intravenous	*in vivo*	Endothelial surface expression of VCAM-1 ↑ (1 - 24 hours after administration)	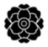	Wagener et al. 2001 [62]
**Von Willebrand factor**	Human	^{59}Fe- and ^{3}H-labeled Hematin	0.25% Na_2CO_3	e.g. 0.17 mg/ml	-	*in vitro*	Formation of FVIII/VWF complex, inhibition of FVIII-VWF dissociation, binding of FVIII/VWF-hematin complex to platelets	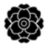	Green et al. 1986 [70]
Von Willebrand factor	Human	Hemin	N/A	10 µM	- (HUVECs)	*in vitro*	Fast Weibel Palade body exocytosis ↑	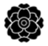	Belcher et al. 2011 [78]
Von Willebrand factor	Human	Hemin	DMSO, diluted in buffer	Molar excess	-	*in vitro*	Protection of FVIII from heme-mediated inactivation	-	Repessé et al. 2012 [71]
Von Willebrand factor	Human	Hemin	50 mM NaOH / 145 mM NaCl, diluted in medium	100 µM	- (HUVECs)	*in vitro*	Weibel Palade body exocytosis ↑, time-dependent VWF secretion ↑ (up to ~65 ng/ml)	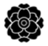	Frimat et al. 2013 [79]
Von Willebrand factor	Human	Hemin (as Panhematin®)	D-sorbitol, Na_2CO_3, saline (as Panhematin®)	20 µM	- (HUVECs)	*in vitro*	Superficial VWF string formation ↑, prevented by hemopexin	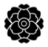	Belcher et al. 2014 [52]

Von Willebrand factor	Mouse (C57BL/6, NY1DD)	Hemin (as Panhematin®)	D-sorbitol, Na_2CO_3, saline (as Panhematin®)	3.2 µmol/kg	intravenous	*in vivo*	VWF expression on vessel walls ↑, dependent on NOX, PKC and oxidants, TLR-4-mediated	✹	Belcher et al. 2014 [52]

💧 = Evidence for a tendency to an anticoagulant effect of heme and its formulations. ✹ = Evidence for a tendency to a procoagulant effect of heme and its formulations. [a] = 10 min after hematin infusion. ECs = endothelial cells; HUVECs = human umbilical vein endothelial cells; ICAM-1 = intercellular adhesion molecule 1; N/A = not applicable; NOX = NADPH oxidase; pdAPC = plasma-derived activated protein C; PKC = protein kinase C; rAPC = recombinant activated protein C (Drotrecogin alpha, Xigris ®) rFVIII = recombinant factor VIII; rFXIIIa = recombinant active factor XIII; TT = thrombin time; VCAM-1 = Vascular cell adhesion molecule 1.

References

1. Brown, W.H. The relation of hematin to pathological pigment formation. *J. Exp. Med.* **1911**, *14*, 612–622.
2. Brown, W.H.; Loevenhart, A.S. The effect of hematin on the circulation and respiration. *J. Exp. Med.* **1913**, *18*, 107–112.
3. Brown, W.H. Malarial pigment (hematin) as an active factor in the production of the blood picture of malaria. *J. Exp. Med.* **1913**, *18*, 96–106.
4. Brown, W.H. The renal complications of hematin intoxification and their relation to Malaria. *Arch. Intern. Med.* **1913**, *XII*, 315–321.
5. Anderson, W.A.D.; Morrison, D.B.; Williams, E.F. Pathologic changes following injections of ferrihemate (hematin) in dogs. *Arch. Pathol.* **1942**, *33*, 589–602.
6. Corcoran, A.C.; Page, I.H. Renal damage from ferroheme pigments myoglobin, hemoglobin, hematin. *Tex. Rep. Biol. Med.* **1945**, *3*, 528–544.
7. Gessler, U.; Loreth, A.; Schröder, K.; Steinhausen, M. Experimentelle Untersuchungen über die glomeruläre Filtration anurischer Ratten nach Hämatinvergiftung. *Klin. Wochenschr.* **1966**, *44*, 628–633.
8. Dhar, G.J.; Bossenmaier, I.; Petryka, Z.J.; Cardinal, R.; Watson, C.J. Effect of hematin in hepatic porphyria: Further studies. *Ann. Intern. Med.* **1975**, *83*, 20–30.
9. Lips, D.L.; Pierach, C.A.; Edwards, P.S. Hematin toxicity in rats. *Toxicol. Lett.* **1978**, *2*, 329–332.
10. Lamon, J.M.; Frykholm, B.C.; Hess, R.A.; Tschudy, D.P. Hematin therapy for acute porphyria. *Medicine (Baltimore).* **1979**, *58*, 252–269.
11. Morris, D.L.; Dudley, M.D.; Pearson, R.D. Coagulopathy associated with hematin treatment for acute intermittent porphyria. *Ann. Intern. Med.* **1981**, *95*, 700–701.
12. McColl, K.E.; Moore, M.R.; Thompson, G.G.; Goldberg, A. Treatment with haematin in acute hepatic porphyria. *Q. J. Med.* **1981**, *50*, 161–174.
13. Bloomer, J.R.; Pierach, C.A. Effect of hematin administration to patients with protoporphyria and liver disease. *Hepatology* **1982**, *2*, 817–821.
14. Mustajoki, P.; Tenhunen, R.; Tokola, O.; Gothoni, G. Haem arginate in the treatment of acute hepatic porphyrias. *Br. Med. J. (Clin. Res. Ed.)* **1986**, *293*, 538–539.

15. Ruutu, T.; Volin, L.; Tenhunen, R. Haem arginate as a treatment for myelodysplastic syndromes. *Br. J. Haematol.* **1987**, *65*, 425–428.
16. Tenhunen, R.; Tokola, O.; Lindén, I.-B. Haem arginate: A new stable haem compound. *J. Pharm. Pharmacol.* **1987**, *39*, 780–786.
17. Simionatto, C.S.; Cabal, R.; Jones, R.L.; Galbraith, R.A. Thrombophlebitis and disturbed hemostasis following administration of intravenous hematin in normal volunteers. *Am. J. Med.* **1988**, *85*, 538–540.
18. Herrick, A.L.; Moore, M.R.; Mccoll, K.E.L.; Cook, A.; Goldberg, A. Controlled trial of haem arginate in acute hepatic porphyria. *Lancet* **1989**, *333*, 1295–1297.
19. Mustajoki, P.; Nordmann, Y. Early administration of heme arginate for acute porphyric attacks. *Arch. Intern Med.* **1993**, *153*, 2004–2008.
20. Gajra, A.; Vajpayee, N.; Singh, M.; Coyle, T.E.; Wright, J. Hematin induced coagulopathy in acute intermittent porphyria: A case report. *Blood* **2000**, *96*, 82b.
21. Anderson, K.E.; Collins, S. Open-label study of hemin for acute porphyria: Clinical practice implications. *Am. J. Med.* **2006**, *119*, 801.e1-801.e6.
22. Marsden, J.T.; Guppy, S.; Stein, P.; Cox, T.M.; Badminton, M.; Gardiner, T.; Barth, J.H.; Stewart, M.F.; Rees, D.C. Audit of the use of regular haem arginate infusions in patients with acute porphyria to prevent recurrent symptoms. *JIMD Rep.* **2015**, *22*, 57–65.
23. Glueck, R.; Green, D.; Cohen, I.; Ts'ao, C. Hematin: Unique effects on hemostasis. *Blood* **1983**, *61*, 243–249.
24. Becker, C.G.; Wagner, M.; Kaplan, A.P.; Silverberg, M.; Grady, R.W.; Liem, H.; Muller-Eberhard, U. Activation of factor XII-dependent pathways in human plasma by hematin and protoporphyrin. *J. Clin. Invest.* **1985**, *76*, 413–419.
25. Jones, R.L. Hematin-derived anticoagulant. Generation in vitro and in vivo. *J. Exp. Med.* **1986**, *163*, 724–739.
26. Volin, L.; Rasi, V.; Vahtera, E.; Tenhunen, R. Heme arginate: effects on hemostasis. *Blood* **1988**, *71*, 625–628.
27. Green, D.; Ts'ao, C. Hematin: Effects on hemostasis. *J. Lab. Clin. Med.* **1990**, *115*, 144–147.
28. Huang, Y.; Komatsu, T.; Nakagawa, A.; Tsuchida, E.; Kobayashi, S. Compatibility in vitro of albumin-heme (O2 carrier) with blood cell components. *J. Biomed. Mater. Res.* **2003**, *66A*, 292–297.
29. Desbuards, N.; Rochefort, G.Y.; Schlecht, D.; Machet, M.-C.; Halimi, J.-M.; Eder, V.; Hyvelin, J.-M.; Antier, D. Heme oxygenase-1 inducer hemin prevents vascular thrombosis. *Thromb. Haemost.* **2007**, *98*, 614–620.
30. Fei, D.; Meng, X.; Zhao, M.M.; Kang, K.A.I.; Tan, G.; Pan, S.; Luo, Y.; Liu, W.E.N.; Nan, C.; Jiang, H.; et al. Enhanced induction of heme oxygenase-1 suppresses thrombus formation and affects the protein C system in sepsis. *Transl. Res.* **2012**, *159*, 99–109.
31. Hassaan, P.S.; Mehanna, R.A.; Dief, A.E. The potential role of hemopexin and heme oxygenase-1 inducer in a model of sepsis. *Physiol. J.* **2015**, *2015*, 1–10.
32. Hopp, M.-T.; Alhanafi, N.; Paul George, A.A.; Hamedani, N.S.; Biswas, A.; Oldenburg, J.; Pötzsch, B.; Imhof, D. Molecular insights and functional consequences of the interaction of heme with activated protein C. *Antioxid. Redox Signal.* **2021**, *34*, 32–48.
33. Rochefort, G.Y.; Libgot, R.; Desbuards, N.; Schlecht, D.; Halimi, J.M.; Ossant, F.; Eder, V.; Antier, D. Effect of the heme oxygenase inducer hemin on blood haemostasis measured by high-frequency ultrasound. *Clin. Exp. Pharmacol. Physiol.* **2007**, *34*, 1272–1275.

34. De Souza, G.R.; Hounkpe, B.W.; Fiusa, M.M.L.; Colella, M.P.; Annichino-Bizzacchi, J.M.; Traina, F.; Costa, F.F.; De Paula, E.V. Tissue factor-dependent coagulation activation by heme: A thromboelastometry study. *PLoS One* **2017**, *12*, 1–10.
35. Goetsch, C.A.; Bissell, D.M. Instability of hematin used in the treatment of acute hepatic porphyria. *N. Engl. J. Med.* **1986**, *315*, 235–238.
36. Barnard, R.D. The effect of saccharin ingestion on blood coagulation and the in vitro anticoagulant effect of saccharin and of ferriheme. *J. Am. Pharm. Assoc. (Scientific ed.)* **1947**, *36*, 225–228.
37. Green, D.; Reynolds, N.; Klein, J.; Kohl, H.; Ts'ao, C.H. The inactivation of hemostatic factors by hematin. *J. Lab. Clin. Med.* **1983**, *102*, 361–9.
38. Peterson, D.; Gerrard, J.; Glover, S.; Rao, G.; White, J. Epinephrine reduction of heme: Implication for understanding the transmission of an agonist stimulus. *Science (80-.).* **1982**, *215*, 71–73.
39. Malik, Z.; Creter, D.; Cohen, A.; Djaldetti, M. Haemin affects platelet aggregation and lymphocyte mitogenicity in whole blood incubations. *Cytobios* **1983**, *38*, 33–38.
40. Neely, S.M.; Gardner, D..; Reynolds, N.; Green, D.; Ts'ao, C. Mechanism and characteristics of platelet activation by haematin. *Br. J. Haematol.* **1984**, *58*, 305–316.
41. Volin, L.; Ruutu, T.; Knuutila, S.; Tenhunen, R. Heme arginate treatment for myelodysplastic syndromes. *Leuk. Res.* **1988**, *12*, 423–431.
42. Peng, L.; Mundada, L.; Stomel, J.M.; Liu, J.J.; Sun, J.; Yet, S.-F.; Fay, W.P. Induction of heme oxygenase-1 expression inhibits platelet-dependent thrombosis. *Antioxid. Redox Signal.* **2004**, *6*, 729–735.
43. Komatsu, T.; Huang, Y.; Wakamoto, S.; Abe, H.; Fujihara, M.; Azuma, H.; Ikeda, H.; Yamamoto, H.; Horinouchi, H.; Kobayashi, K.; et al. Influence of O2-carrying plasma hemoprotein "albumin-heme" on complement system and platelet activationin vitro and physiological responses to exchange transfusion. *J. Biomed. Mater. Res. Part A* **2007**, *81A*, 821–826.
44. Woollard, K.J.; Sturgeon, S.; Chin-Dusting, J.P.F.; Salem, H.H.; Jackson, S.P. Erythrocyte hemolysis and hemoglobin oxidation promote ferric chloride-induced vascular injury. *J. Biol. Chem.* **2009**, *284*, 13110–13118.
45. NaveenKumar, S.K.; SharathBabu, B.N.; Hemshekhar, M.; Kemparaju, K.; Girish, K.S.; Mugesh, G. The role of reactive oxygen species and ferroptosis in heme-mediated activation of human platelets. *ACS Chem. Biol.* **2018**, *13*, 1996–2002.
46. NaveenKumar, S.K.; Hemshekhar, M.; Kemparaju, K.; Girish, K.S. Hemin-induced platelet activation and ferroptosis is mediated through ROS-driven proteasomal activity and inflammasome activation: Protection by melatonin. *Biochim. Biophys. Acta - Mol. Basis Dis.* **2019**, *1865*, 2303–2316.
47. Bourne, J.H.; Colicchia, M.; Di, Y.; Martin, E.; Slater, A.; Roumenina, L.T.; Dimitrov, J.D.; Watson, S.P.; Rayes, J. Heme induces human and mouse platelet activation through C-type-lectin-like receptor-2. *Haematologica* **2020**.
48. Neely, S.M.; Gardner, D. V; Green, D.; Ts'ao, C.H. Effect of hematin on endothelial cells and endothelial cell-platelet interactions. *Am. J. Pathol.* **1984**, *115*, 390–396.
49. Balla, G.; Vercollotti, G.; Muller-Eberhard, U.; Eaton, J.; Jacob, H.S.; Vercellotti, G.M.; Muller-Eberhard, U.; Eaton, J.; Jacob, H.S. Exposure of endothelial cells to free heme potentiates damage mediated by granulocytes and toxic oxygen species. *Lab. Investig.* **1991**, *64*, 648–655.

50. Balla, G.; Jacob, H.S.; Eaton, J.W.; Belcher, J.D.; Vercellotti, G.M. Hemin: A possible physiological mediator of low density lipoprotein oxidation and endothelial injury. *Arterioscler. Thromb. A J. Vasc. Biol.* **1991**, *11*, 1700–1711.
51. Higdon, A.N.; Benavides, G.A.; Chacko, B.K.; Ouyang, X.; Johnson, M.S.; Landar, A.; Zhang, J.; Darley-Usmar, V.M. Hemin causes mitochondrial dysfunction in endothelial cells through promoting lipid peroxidation: The protective role of autophagy. *Am. J. Physiol. Circ. Physiol.* **2012**, *302*, H1394–H1409.
52. Belcher, J.D.; Chen, C.; Nguyen, J.; Milbauer, L.; Abdulla, F.; Alayash, A.I.; Smith, A.; Nath, K.A.; Hebbel, R.P.; Vercellotti, G.M.; et al. Heme triggers TLR4 signaling leading to endothelial cell activation and vaso-occlusion in murine sickle cell disease. *Blood* **2014**, *123*, 377–390.
53. Camus, S.M.; De Moraes, J.A.; Bonnin, P.; Abbyad, P.; Le Jeune, S.; Lionnet, F.; Loufrani, L.; Grimaud, L.; Lambry, J.-C.; Charue, D.; et al. Circulating cell membrane microparticles transfer heme to endothelial cells and trigger vasoocclusions in sickle cell disease. *Blood* **2015**, *125*, 3805–3814.
54. Singla, S.; Sysol, J.R.; Dille, B.; Jones, N.; Chen, J.; Machado, R.F. Hemin causes lung microvascular endothelial barrier dysfunction by necroptotic cell death. *Am. J. Respir. Cell Mol. Biol.* **2017**, *57*, 307–314.
55. May, O.; Merle, N.S.; Grunenwald, A.; Gnemmi, V.; Leon, J.; Payet, C.; Robe-Rybkine, T.; Paule, R.; Delguste, F.; Satchell, S.C.; et al. Heme drives susceptibility of glomerular endothelium to complement overactivation due to inefficient upregulation of heme oxygenase-1. *Front. Immunol.* **2018**, *9*, 3008.
56. Merle, N.S.; Grunenwald, A.; Rajaratnam, H.; Gnemmi, V.; Frimat, M.; Figueres, M.L.; Knockaert, S.; Bouzekri, S.; Charue, D.; Noe, R.; et al. Intravascular hemolysis activates complement via cell-free heme and heme-loaded microvesicles. *JCI insight* **2018**.
57. Kucukal, E.; Ilich, A.; Key, N.S.; Little, J.A.; Gurkan, U.A. Red blood cell adhesion to heme-activated endothelial cells reflects clinical phenotype in sickle cell disease. *Am. J. Hematol.* **2018**, *93*, 1050–1060.
58. Chou, A.C.; Fitch, C.D. Mechanism of hemolysis induced by ferriprotoporphyrin IX. *J. Clin. Invest.* **1981**, *68*, 672–677.
59. Liu, S.C.; Zhai, S.; Lawler, J.; Palek, J. Hemin-mediated dissociation of erythrocyte membrane skeletal proteins. *J. Biol. Chem.* **1985**, *260*, 12234–9.
60. Shaklai, N.; Shviro, Y.; Rabizadeh, E.; Kirschner-Zilber, I. Accumulation and drainage of hemin in the red cell membrane. *Biochim. Biophys. Acta - Biomembr.* **1985**, *821*, 355–366.
61. Smith, D.J.; Winslow, R.M. Effects of extraerythrocytic hemoglobin and its components on mononuclear cell procoagulant activity. *J. Lab. Clin. Med.* **1992**, *119*, 176–182.
62. Wagener, F.A.D.T.G.; Eggert, A.; Boerman, O.C.; Oyen, W.J.G.; Verhofstad, A.; Abraham, N.G.; Adema, G.; Van Kooyk, Y.; De Witte, T.; Figdor, C.G. Heme is a potent inducer of inflammation in mice and is counteracted by heme oxygenase. *Blood* **2001**, *98*, 1802–1811.
63. Graça-Souza, A. V.; Arruda, M.A.B.; De Freitas, M.S.; Barja-Fidalgo, C.; Oliveira, P.L. Neutrophil activation by heme: Implications for inflammatory processes. *Blood* **2002**, *99*, 4160–4165.
64. Wagener, F.A.D.T.G.; van Beurden, H.E.; von den Hoff, J.W.; Adema, G.J.; Figdor, C.G. The heme-heme oxygenase system: A molecular switch in wound healing. *Blood* **2003**, *102*, 521–528.

65. Arruda, M.A.; Rossi, A.G.; de Freitas, M.S.; Barja-Fidalgo, C.; Graça-Souza, A. V. Heme inhibits human neutrophil apoptosis: Involvement of phosphoinositide 3-kinase, MAPK, and NF-κB. *J. Immunol.* **2004**, *173*, 2023–2030.
66. Chen, G.; Zhang, D.; Fuchs, T.A.; Manwani, D.; Wagner, D.D.; Frenette, P.S. Heme-induced neutrophil extracellular traps contribute to the pathogenesis of sickle cell disease. *Blood* **2014**, *123*, 3818–3827.
67. Ohbuchi, A.; Kono, M.; Kitagawa, K.; Takenokuchi, M.; Imoto, S.; Saigo, K. Quantitative analysis of hemin-induced neutrophil extracellular trap formation and effects of hydrogen peroxide on this phenomenon. *Biochem. Biophys. Reports* **2017**, *11*, 147–153.
68. Arkebauer, M.R.; Kanaparthy, S.S.; Malayaman, S.N.; Vosseller, K.; Nielsen, V.G. Carbon monoxide and nitric oxide modulate α2-antiplasmin and plasmin activity. *Blood Coagul. Fibrinolysis* **2011**, *22*, 712–719.
69. Vijayan, V.; Wagener, F.A.D.T.G.; Immenschuh, S. The macrophage heme-heme oxygenase-1 system and its role in inflammation. *Biochem. Pharmacol.* **2018**, *153*, 159–167.
70. Green, D.; Furby, F.H.; Berndt, M.C. The interaction of the VIII/von Willebrand factor complex with hematin. *Thromb. Haemost.* **1986**, *56*, 277–282.
71. Repessé, Y.; Dimitrov, J.D.; Peyron, I.; Moshai, E.F.; Kiger, L.; Dasgupta, S.; Delignat, S.; Marden, M.C.; Kaveri, S. V.; Lacroix-Desmazes, S. Heme binds to factor VIII and inhibits its interaction with activated factor IX. *J. Thromb. Haemost.* **2012**, *10*, 1062–1071.
72. Sparkenbaugh, E.M.; Chantrathammachart, P.; Wang, S.; Jonas, W.; Kirchhofer, D.; Gailani, D.; Gruber, A.; Kasthuri, R.; Key, N.S.; Mackman, N.; et al. Excess of heme induces tissue factor-dependent activation of coagulation in mice. *Haematologica* **2015**, *100*, 308–313.
73. Nielsen, V.G.; Cohen, J.B.; Malayaman, S.N.; Nowak, M.; Vosseller, K. Fibrinogen is a heme-associated, carbon monoxide sensing molecule: A preliminary report. *Blood Coagul. Fibrinolysis* **2011**, *22*, 443–447.
74. Orino, K. Functional binding analysis of human fibrinogen as an iron- and heme-binding protein. *BioMetals* **2013**, *26*, 789–794.
75. Ke, Z.; Huang, Q. Haem-assisted dityrosine-cross-linking of fibrinogen under non-thermal plasma exposure: One important mechanism of facilitated blood coagulation. *Sci. Rep.* **2016**, *6*, 1–8.
76. Hou, T.; Zhang, Y.; Wu, T.; Wang, M.; Zhang, Y.; Li, R.; Wang, L.; Xue, Q.; Wang, S. Label-free detection of fibrinogen based on fibrinogen-enhanced peroxidase activity of fibrinogen-hemin composite. *Analyst* **2018**, *143*, 725–730.
77. Musser, D.A.; Wagner, J.M.; Weber, F.J.; Datta-Gupta, N. The binding of tumor localizing porphyrins to a fibrin matrix and their effects following photoirradiation. *Res. Commun. Chem. Pathol. Pharmacol.* **1980**, *28*, 505–25.
78. Belcher, J.D.; Nguyen, J.; Chen, C.; Smith, A.; Alayash, A.I.; Nair, S.L.; Somani, A.; Hebbel, R.P.; Vercellotti, G.M. Plasma hemoglobin and heme trigger Weibel Palade body exocytosis and vaso-occlusion in transgenic sickle mice. *Blood* **2011**, *118*, 896–896.
79. Frimat, M.; Tabarin, F.; Dimitrov, J.D.; Poitou, C.; Halbwachs-Mecarelli, L.; Fremeaux-Bacchi, V.; Roumenina, L.T. Complement activation by heme as a secondary hit for atypical hemolytic uremic syndrome. *Blood* **2013**, *122*, 282–292.

80. Jones, R.L. Hematin-derived anticoagulant retards fibrin formation and is inhibited by iron chelators. *Ann. New York Acad. Sci.* **1986**, *485*, 418–420.

81. Setty, B.N.Y.; Betal, S.G.; Zhang, J.; Stuart, M.J. Heme induces endothelial tissue factor expression: Potential role in hemostatic activation in patients with hemolytic anemia. *J. Thromb. Haemost.* **2008**, *6*, 2202–2209.

82. Rehani, T.; Mathson, K.; Belcher, J.D.; Vercellotti, G.M.; Slungaard, A. Heme potently stimulates tissue factor expression by peripheral blood monocytes: A novel mechanism for thrombosis in intravascular hemolytic diseases. *Blood* **2013**, *122*, 2215.

83. Souza, G.R.; Fiusa, M.M.L.; Lanaro, C.; Colella, M.P.; Montalvao, S.A.L.; Saad, S.T.O.; Costa, F.F.; Traina, F.; Annichino-Bizzacchi, J.M.; De Paula, E.V. Coagulation activation by heme: Evidence from global hemostasis assays. *Blood* **2014**, *124*, 455–455.

84. Hounkpe, B.W.; Moraes, C.R.P.; do Santos, M.N.N.; Costas, F.F.; De Paula, E. V. Heme induces mRNA expression and activation of tissue factor by TLR4 dependent mechanisms. *medRxiv* **2020**.

85. May, O.; Yatime, L.; Merle, N.S.; Delguste, F.; Howsam, M.; Daugan, M. V.; Paul-Constant, C.; Billamboz, M.; Ghinet, A.; Lancel, S.; et al. The receptor for advanced glycation end products is a sensor for cell-free heme. *FEBS J.* **2020**.
